# Supplementary material for: Learning the distribution of single-cell chromosome conformations in bacteria reveals emergent order across genomic scales
Source: Nat Commun. 2021 Mar 30;12:1963. doi: 10.1038/s41467-021-22189-x (PMC8010069; doi:10.1038/s41467-021-22189-x)
Supplement: Supplementary file 1 — Supplementary Information [file 41467_2021_22189_MOESM1_ESM.pdf]

Supplementary Information

Learning the distribution of single-cell chromosome conformations in bacteria reveals emergent order across genomic scales

J.J.B. Messelink et al.

Contents

1 Experimental procedures on *C. crescentus* cells 3
1.1 Experimental determination of distances between loci 10 kb apart 3
1.2 Determination of chromosome density via SIM microscopy 3

2 Data analysis: using experimental distance distributions to set the coarse-grained representation of the lattice polymer 9
2.1 Analysis of experimental distance distributions of pairs of loci in *C. crescentus* . . . 9
2.2 Setting the dimensions of the lattice spacing and the cellular confinement in the model 11

3 Inverse Monte Carlo algorithm for MaxEnt chromosome model 12
3.1 Forward algorithm 13
3.2 Inverse algorithm 14
3.3 Ergodicity of forward algorithm 15

4 Testing the inverse Monte Carlo algorithm 17

5 Hi-C data filtering 17
5.1 DNA replication inhibited Hi-C datasets 18
5.2 Filter procedure 18

6 Comparison of filter procedure for wild-type replicates 21

7 Results for MaxEnt model trained on unfiltered Hi-C data 22

8 Results for MaxEnt model trained on replication-inhibited cells 27
8.1 DnaA-depleted cells 27
8.2 Cells overexpressing *ctrA(D51E)*$\Delta 3\Omega$ 28

9 Analysis of genomic Super Domains 32
9.1 Super Domain definition and long-axis exclusion analysis 32
9.2 Super Domain properties 32

10 Overlap analysis between local chromosome extension peaks and highly transcribed genes 35

11 Relation between Hi-C scores and average distance and distance correlations 37

12 A global rotation does not produce the observed long-axis correlation pattern 38

13 MaxEnt models for Δ*smc* cells and rifampicin-treated cells 39

14 Estimates of localization information 43

15 Local extension interval and origin of *ori* and *ter* extensions 43

16 Linear spatial organization of a polymer with juxtaposed chromosomal arms 44

17 Independence of results for modified MaxEnt models 46

1 Experimental procedures on *C. crescentus* cells

Bacterial strains and growth conditions

All *C. crescentus* strains used in this study were derived from the synchronizable wild-type CB15N (NA1000). Cells were grown in peptone-yeast extract (PYE) medium (Pointdexter, 1964) at 28°C under aerobic conditions (shaking at 210 rpm). When appropriate, the medium was supplemented with antibiotics at the following concentrations (μg ml^{-1} in liquid/solid medium): kanamycin (30/50), gentamicin (15/20), and spectinomycin (50/100).

1.1 Experimental determination of distances between loci 10 kb apart

Plasmid and strain construction

To measure the distances between chromosomal loci that are located 10 kb apart, *C. crescentus* strains were constructed whose chromosomes contained binding sites for fluorescently tagged DNA binding proteins. The bacterial strains, plasmids, and oligonucleotides used in this study are listed in Tables S1-S4. *Escherichia coli* TOP10 (Invitrogen) was used as host for cloning purposes. All plasmids were verified by DNA sequencing. Plasmids carrying 10 copies of either *lac*O (PCR-amplified from plasmid pLAU43 [1]) or *tetO* (PCR-amplified from plasmid pLAU44 [1]) were transferred to *C. crescentus* by electroporation [2] and integrated at various chromosomal loci by single-homologous recombination. Subsequently, a two-gene operon encoding LacI-eCFP and TetR-eYFP was integrated at the *xylX* locus by phiCr30-mediated phage transduction [2], using a lysate of a strain transformed with plasmid pHPV472 [3]. Proper chromosomal integration was verified by colony PCR.

Measurement of distance between pairs of loci 10 kb apart

All microscopy analyses to determine the distance between chromosomal loci were performed on cells grown in PYE medium containing kanamycin and gentimicin to the mid-exponential phase (OD 0.4), and subsequently synchronized [4]. Immediately after synchronization, swarmer cells were immobilized on pads made of 1% agarose in PYE medium. Cells were observed with a Zeiss Axio Observer.Z1 microscope equipped with an alpha Plan-Apochromat 100x/1.46 Oil Ph3 objective (Zeiss, Germany). An X-Cite 120PC metal halide light source (EXFO, Canada), combined with ET-CFP and ET-YFP filter cubes (Chroma, USA), was used for the detection of fluorescent foci. Images were taken with a pco.edge sCMOS camera (pco, Germany) and recorded with VisiView 2.1.4 (Visitron, Germany). To identify the subpixel localization of the fluorescent foci, a 2D Gaussian was fitted to each fluorescent focus using the GDSC SMLM plugin [5] [1] for ImageJ2 [6]. In order to correct for systematic shifts between the YFP and CFP channels, fiducials (Tetraspeck microspheres, 0.5 μm, Invitrogen/Thermo Fischer Scientific, USA) were imaged in the YFP and CFP channels and analyzed with the same set-up and pipeline.

1.2 Determination of chromosome density via SIM microscopy

In order to investigate the intracellular distribution of the chromosome, *C. crescentus* wild-type cells were grown and synchronized as described above. Immediately after synchronization, the cells

[1] http://www.sussex.ac.uk/gdsc/intranet/microscopy/UserSupport/AnalysisProtocol/imagej/smlm_plugins/

were incubated with 0.5 μg/ml of the DNA-stain DAPI (4',6-diamino-2-phenylindole) for 5 min at 28°C. Cells were then washed (5 min at 4000 g), resuspended in M2 salts buffer [2] and applied to pads made of 1% agarose in water, before they were imaged with a Zeiss Elyra 7 Lattice SIM microscope equipped with an alpha Plan-Apochromat 100x/1.46 Oil Objective (Zeiss, Germany). DAPI was excited with a 405 nm laser and its emission was recorded in the 420-480 nm range.

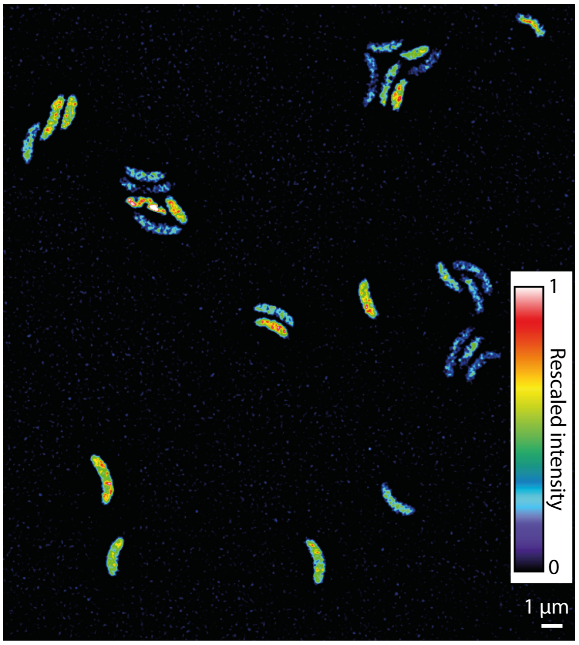

Supplementary Figure 1: SIM microscopy image example SIM microscopy image of a single focal plane out of a z-stack shows the DAPI-stained DNA inside multiple *C. crescentus* cells immediately after synchronization. The DNA is organized in a heterogeneous fashion, with several regions of high-density chromosome packing per cell, and shows a clear cell-to-cell variation. The intensity is rescaled for the entire image, such that the highest measured intensity is 1, and the lowest is 0. Scale bar: 1 μm. Shown is a representative image of one of the two biological replicates, which both showed similar results.

Supplementary Table 1: Strains used in this study.

Strain	Genotype/description	Construction/Reference
***E. coli* strains**		
TOP10	Cloning strain	Invitrogen
***C. crescentus* strains**		
CB15N	Synchronizable wild-type strain	Evinger & Agabian (1977) [7]
MvT151	CB15N P_{xyl}::P_{xyl}-*lacI-ecfp-tetR-eyfp* 10x *tetO* and 10x *lacO* spaced 10.0 kb apart at 196°	Consecutive integration of pMvT149, pMvT150 and P_{xyl}-*lacI-ecfp-tetR-eyfp* into CB15N
MvT152	CB15N P_{xyl}::P_{xyl}-*lacI-ecfp-tetR-eyfp* 10x *tetO* and 10x *lacO* spaced 10.1 kb apart at 212°	Consecutive integration of pMvT151, pMvT152 and P_{xyl}-*lacI-ecfp-tetR-eyfp* into CB15N
MvT170	CB15N P_{xyl}::P_{xyl}-*lacI-ecfp-tetR-eyfp* 10x *tetO* and 10x *lacO* spaced 10.1 kb apart at 21°	Consecutive integration of pMvT161, pMvT162 and P_{xyl}-*lacI-ecfp-tetR-eyfp* into CB15N
MvT171	CB15N P_{xyl}::P_{xyl}-*lacI-ecfp-tetR-eyfp* 10x *tetO* and 10x *lacO* spaced 10.0 kb apart at 108°	Consecutive integration of pMvT163, pMvT164 and P_{xyl}-*lacI-ecfp-tetR-eyfp* into CB15N
MvT172	CB15N P_{xyl}::P_{xyl}-*lacI-ecfp-tetR-eyfp* 10x *lacO* and 10x *tetO* spaced 10.0 kb apart at 108°	Consecutive integration of pMvT165, pMvT166 and P_{xyl}-*lacI-ecfp-tetR-eyfp* into CB15N
MvT179	CB15N P_{xyl}::P_{xyl}-*lacI-ecfp-tetR-eyfp* 10x *tetO* and 10x *lacO* spaced 10.1 kb apart at 311°	Consecutive integration of pMvT159, pMvT160 and P_{xyl}-*lacI-ecfp-tetR-eyfp* into CB15N

Supplementary Table 2: Plasmids used in this study.

Plasmid	Description	Reference
**Basic vectors**		
pLAU43	Plasmid carrying 240 LacI binding sites (*lacO*), Kan^R	Lau et al., 2003 [1]
pLAU44	Plasmid carrying 240 TetO binding sites (*tetO*), Gen^R	Lau et al., 2003 [1]
pHPV472	Plasmid carrying P_{xyl}-*lacI-ecfp tetR-eyfp*, Spc^R Str^R	Viollier et al., 2004 [3]
pMCS-2	Integrating plasmid containing multiple cloning site, Kan^R	Thanbichler et al., 2007 [8]
pMCS-4	Integrating plasmid containing multiple cloning site, Gen^R	Thanbichler et al., 2007 [8]
**Plasmids constructed in this work**		
pMvT149	pMCS-2 including 10x *tetO* and part of CCNA_02049, Kan^R	This study
pMvT150	pMCS-4 including 10x *lacO* and part of a chromosomal fragment close to CCNA_02054, Gen^R	This study
pMvT151	pMCS-2 including 10x *tetO* and part of a chromosomal fragment close to CCNA_02228, Kan^R	This study
pMvT152	pMCS-4 including 10x *lacO* and part of a chromosomal fragment close to CCNA_02233, Gen^R	This study
pMvT159	pMCS-2 including 10x *tetO* and part of CCNA_03310, Kan^R	This study
pMvT160	pMCS-4 including 10x *lacO* and part of a chromosomal fragment close to CCNA_03317, Gen^R	This study
pMvT161	pMCS-2 including 10x *tetO* and part of CCNA_00217, Kan^R	This study
pMvT162	pMCS-4 including 10x *lacO* and part of a chromosomal fragment close to CCNA_00226, Gen^R	This study
pMvT163	pMCS-2 including 10x *tetO* and part of CCNA_01105, Kan^R	This study
pMvT164	pMCS-4 including 10x *lacO* and part of a chromosomal fragment close to CCNA_01112, Gen^R	This study
pMvT165	pMCS-2 including 10x *lacO* and part of CCNA_01105, Kan^R	This study
pMvT166	pMCS-4 including 10x *tetO* and part of a chromosomal fragment close to CCNA_01112, Gen^R	This study

Supplementary Table 3: Construction of plasmids.

Plasmid	Description
pMvT149	a) amplification of 10 *tetO* motifs from pLAU44 using oMvT789 & oMvT790 (product 433 bp) and 800 bp from NA1000 gDNA using oMvT791 & oMvT792 (product 848 bp)
	b) fusion of two inserts with pMCS-2/NdeI+NheI via Gibson Assembly
pMvT150	a) amplification of 10 *lacO* motifs from pLAU43 using oMvT796 & oMvT797 (product 547 bp) and 800 bp from NA1000 gDNA using oMvT798 & oMvT799 (product 845 bp)
	b) fusion of two inserts with pMCS-2/NdeI+NheI via Gibson Assembly
pMvT151	a) amplification of 10 *tetO* motifs from pLAU44 using oMvT803 & oMvT804 (product 435 bp) and 800 bp from NA1000 gDNA using oMvT805 & oMvT806 (product 843 bp)
	b) fusion of two inserts with pMCS-2/NdeI+NheI via Gibson Assembly
pMvT152	a) amplification of 10 *lacO* motifs from pLAU43 using oMvT808 & oMvT809 (product 548 bp) and 800 bp from NA1000 gDNA using oMvT810 & oMvT811 (product 845 bp)
	b) fusion of two inserts with pMCS-2/NdeI+NheI via Gibson Assembly
pMvT159	a) amplification of 10 *tetO* motifs from pLAU44 using oMvT789 & oMvT839 (product 435 bp) and 800 bp from NA1000 gDNA using oMvT840 & oMvT841 (product 843 bp)
	b) fusion of two inserts with pMCS-2/NdeI+NheI via Gibson Assembly
pMvT160	a) amplification of 10 *lacO* motifs from pLAU43 using oMvT819 & oMvT842 (product 549 bp) and 800 bp from NA1000 gDNA using oMvT843 & oMvT844 (product 851 bp)
	b) fusion of two inserts with pMCS-2/NdeI+NheI via Gibson Assembly
pMvT161	a) amplification of 10 *tetO* motifs from pLAU44 using oMvT789 & oMvT849 (product 436 bp) and 800 bp from NA1000 gDNA using oMvT850 & oMvT851 (product 840 bp)
	b) fusion of two inserts with pMCS-2/NdeI+NheI via Gibson Assembly
pMvT162	a) amplification of 10 *lacO* motifs from pLAU43 using oMvT819 & oMvT854 (product 549 bp) and 800 bp from NA1000 gDNA using oMvT855 & oMvT856 (product 844 bp)
	b) fusion of two inserts with pMCS-2/NdeI+NheI via Gibson Assembly
pMvT163	a) amplifation of 10 *tetO* motifs from pLAU44 using oMvT789 & oMvT859 (product 436 bp) and 800 bp from NA1000 gDNA using oMvT860 & oMvT861 (product 848 bp)
	b) fusion of two inserts with pMCS-2/NdeI+NheI via Gibson Assembly
pMvT164	a) amplification of 10 *lacO* motifs from pLAU43 using oMvT819 & oMvT863 (product 547 bp) and 800 bp from NA1000 gDNA using oMvT864 & oMvT865 (product 851 bp)
	b) fusion of two inserts with pMCS-2/NdeI+NheI via Gibson Assembly
pMvT165	a) amplification of 10 *lacO* motifs from pLAU43 using oMvT819 & oMvT867 (product 548 bp) and 800 bp from NA1000 gDNA using oMvT868 & oMvT861 (product 845 bp)
	b) fusion of two inserts with pMCS-2/NdeI+NheI via Gibson Assembly
pMvT166	a) amplification of 10 *tetO* motifs from pLAU44 using oMvT789 & oMvT869 (product 435 bp) and 800 bp from NA1000 gDNA using oMvT870 & oMvT865 (product 848 bp)
	b) fusion of two inserts with pMCS-2/NdeI+NheI via Gibson Assembly

Supplementary Table 4: Oligonucliotides used in this study.

ID	Name	Sequence (5' to 3')
oMvT789	tetO _CCNA_02049_p1	cgagacgtccaattgcatatgtccctatcagtgatagagaggggaaagg
oMvT790	tetO _CCNA_02049_p2	cgccgctggccaccggatctctatcactgatagggaccttcccttctg
oMvT791	tetO _CCNA_02049_p3	gggaaggtccctatcagtgatagagatccggtggccagcggcgaac
oMvT792	tetO _CCNA_02049_p4	gatcccccgggctgcagctagcgcgcactgaggccgatggcg
oMvT796	lacO _CCNA_02049_p1	gcgagacgtccaattgcatatgttgtgagcggataacaattggagcaag
oMvT797	lacO _CCNA_02049_p2	cttcgaccgctgggacttcttgttatccgctcacaatttgccttttgc
oMvT798	lacO _CCNA_02049_p3	ggcaaattgtgagcggataacaagaagtcccagcggtcgaagaggacg
oMvT799	lacO _CCNA_02049_p4	gatcccccgggctgcagctagcgcctatgacgtgatgagctccaagcac
oMvT803	tetO _CCNA_02228_p1	cgagacgtccaattgcatatgtccctatcagtgatagagaggggaaagg
oMvT804	tetO _CCNA_02228_p2	gacgacccccctactggtcctctctatcactgatagggaccttccc
oMvT805	tetO _CCNA_02228_p3	ggtccctatcagtgatagagaggaccagtaggggggtcgtcgaacg
oMvT806	tetO _CCNA_02228_p4	gatcccccgggctgcagctagcccagccccgccgccgacatcg
oMvT808	lacO _CCNA_02228_p1	gcgagacgtccaattgcatatgttgtgagcggataacaattggagcaag
oMvT809	lacO _CCNA_02228_p2	cccaggcaacttgtctttcgttgttatccgctcacaatttgccttttgc
oMvT810	lacO _CCNA_02228_p3	ggcaaattgtgagcggataacaacgaaagacaagttgcctgggc
oMvT811	lacO _CCNA_02228_p4	gatcccccgggctgcagctagcctagcggatcgggcgcgcgaag
oMvT819	lacO _CCNA_01737_p1	gcgagacgtccaattgcatatgttgtgagcggataacaattggagcaag
oMvT839	tetO _nusG_p2	ggtcgaaaagatcgcctgatctctatcactgatagggaccttcccttc
oMvT840	tetO _nusG_p3	ggtccctatcagtgatagagatcaggcgatcttttcgacctgattg
oMvT841	tetO _nusG_p4	gatcccccgggctgcagctagccgcgacagccgccgccgctcc
oMvT842	lacO _CC-3211_p2	gcagccgcgatttccattgagttgttatccgctcacaatttgccttttg
oMvT843	lacO _CC-3211_p3	ggcaaattgtgagcggataacaactcaatggaaatcgcggctgcgg
oMvT844	lacO _CC-3211_p4	ctagtggatcccccgggctgcagctagcctgccaggagacgcggcc
oMvT849	tetO _CC-0217_p2	cagcgcatagcccagcgcgctctctatcactgatagggaccttcccttc
oMvT850	tetO _CC-0217_p3	ggtccctatcagtgatagagagcgcgctgggctatgcgctgac
oMvT851	tetO _CC-0217_p4	cccccgggctgcagctagcctagctccccgccctctcgatcg
oMvT854	lacO _CC-0226_p2	caactatgtcgatgacgagcattgttatccgctcacaatttgccttttg
oMvT855	lacO _CC-0226_p3	caaattgtgagcggataacaatgctcgtcatcgacatagttgctgcg
oMvT856	lacO _CC-0226_p4	ggatcccccgggctgcagctagcgtgatgaccaagaccatgcttctggc
oMvT859	tetO _CC-1053_p2	gcccagatgccggcgcaatctctctatcactgatagggaccttcccttc
oMvT860	tetO _CC-1053_p3	gggaaggtccctatcagtgatagagagattgcgccggcatctgggcc
oMvT861	tetO _CC-1053_p4	gatcccccgggctgcagctagcggcaggatcgaccaccgcgc
oMvT863	lacO _CC-1059_p2	ccagttcgcagagccggcgttgttatccgctcacaatttgccttttgc
oMvT864	lacO _CC-1059_p3	caaaaggcaaattgtgagcggataacaacgccggctctgcgaactggag
oMvT865	lacO _CC-1059_p4	ggatcccccgggctgcagctagctcatgccatccggtagtgtcgggc
oMvT867	lacO _CC-1053_p2	gcccagatgccggcgcaatcttgttatccgctcacaatttgccttttgc
oMvT868	lacO _CC-1053_p3	ggcaaattgtgagcggataacaagattgcgccggcatctgggc
oMvT869	tetO _CC-1059_p2	ccagttcgcagagccggcgtctctatcactgatagggaccttcccttc
oMvT870	tetO _CC-1059_p3	ggaaggtccctatcagtgatagagacgccggctctgcgaactggag

2 Data analysis: using experimental distance distributions to set the coarse-grained representation of the lattice polymer

We require a coarse-grained representation of the bacterial chromosome that is consistent with experimentally determined statistics beyond the coarse-graining length scale. Furthermore, our coarse-grained representation should allow for efficient computation. The resolution of the Hi-C data set (10 kb) sets a natural coarse-graining scale for the polymer, but we require additional experiments for the statistics at this length-scale: the distribution of spatial distances between pairs of loci at a 10 kb genomic distance. Here we demonstrate that a lattice polymer representation of the chromosome captures the statistics at this length scale. In this representation, the measured average spatial distance between a pair of loci sets the lattice spacing of our representation of the bacterial chromosome.

2.1 Analysis of experimental distance distributions of pairs of loci in *C. crescentus*

From the experimental procedure described in Note 1, a data set of 100 2D distance vectors are obtained in *C. crescentus* for five pairs of loci separated by 10 kb. Note, microscopy data only gives us the projected 2D distances, while the actual distance vectors are in 3D. From the 2D data set, however, we can infer the underlying distribution of 3D distances. To make this inference, two effects are considered:

1. Measurement errors. This has two sources: finite localization precision and drift between the two consecutive images, taken to determine the positions of the two fluorescently (YFP and CFP) labeled loci using two different fluorescence channels.

The measurement noise due to finite localization precision depends on the intensity of the fluorescent probe and the brightness of its direct surroundings. We calculated this precision using the GDSC SMLM plugin to have a standard error of 32.63 nm, with an average variation between measurements of 0.02 nm.

To account for drift between two consecutive images, we decompose the distance vector within each pair of foci into an x and y component, and sum these two components separately for all cells. As the orientations of cells are isotropically distributed, both the x and y component sums should go to 0 for increasing sample size. However, we find significant deviations from 0, larger than expected with our finite sampling, indicating a systematic drift estimated to be 35 ± 4nm in the x-direction, and 52 ± 5nm in the y-direction (error on the mean). We correct for these deviations by subtracting the systematic drift in the x and y directions from each of the experimentally measured distance vectors, from which a model for the 3D distance distribution is inferred. This correction will, however, be an overestimate: for a finite sample size, the x and y component sums will likely deviate from 0, even in the absence of drift. To account for this bias in the drift estimator, we simulate finite sampling of 2D distance vectors (using the same number of data points as in the experiments) from the inferred model for the 3D distance distribution. Note, we require a self-consistent iterative procedure: the bias in the drift estimator that we correct for, when inferring the 3D model from measured 2D distances, must be consistent with the bias we determine when performing a finite sampling of 2D distances from this model.

2. We consider intrinsic variations in 3D distances between the loci, for instance due to thermal fluctuations of the DNA. We assume that the underlying distribution of relative positions is de-

Supplementary Table 5: Inferred average distances for the measured pairs of loci. The data sets MvT171 and MvT172 are for the same loci, just with their markers switched (see Note 1). The determined distances for each of these pairs are within two standard deviations of each other.

Data set	Average 3D distance (nm)	Inferred σ (nm)
MvT151	106 ± 7	67
MvT170	134 ± 8	84
MvT171	121 ± 8	76
MvT152	158 ± 9	99
MvT172	132 ± 8	83
MvT179	124 ± 7	78
**Inferred average for entire chromosome**	Average 3D distance (nm)	Variance (nm)
	129 ± 7	17

scribed by a 3D Gaussian with a standard deviation and a mean equal to 0. This results in one fit parameter (σ) for the underlying distribution.

To determine the value of σ for each of the pairs of loci, we also use an iterative procedure: we start by choosing an initial value of σ, and then simulate the sampling of a large number of 3D distance vectors from this distribution. We then take a 2D projection of these samples and add the random measurement error of 32.63 nm (see point 1). Next, we compute the average 2D distance and compare with the experimentally determined 2D average distance. If these values are not equal, the value of σ is updated accordingly, and a new round of the iteration begins. This procedure is repeated until convergence is reached (the average 2D distance is equal to the experimentally determined 2D average distance).

Once convergence is reached, the mean 3D distance for each pair of loci is calculated through a forward simulation of random points being drawn from a 3D Gaussian. The error on the mean inferred 3D distance for a specific pair of loci on the chromosome is determined by bootstrapping (see Table 5). The average distance for the entire chromosome is taken as the average over the means of the 5 pairs of loci we studied experimentally, and is determined to be 129 ± 7 nm (standard error of the mean).

Once the average distances are matched between model and experiment, the distributions of measured distances can also be compared. This distribution matches well between model and experiment (Supplementary Fig. 2), supporting the assumption of a 3D gaussian as an underlying distribution of relative positions between the loci. Once we set the lattice constant of our lattice polymer to match this average 3D distance, our lattice polymer model approximately captures the correct statistics for the distance between neighboring chromosomal regions. This validates the use of a lattice polymer to connect consecutive monomers representing neighboring chromosomal regions.

2.2 Setting the dimensions of the lattice spacing and the cellular confinement in the model

We employ a polymer model on a cubic lattice. In this representation, the position of each fourth monomer indicates the unit cell occupied by the center of a Hi-C chromosomal region. The polymer model is allowed to intersect, since multiple centers of genomic regions could reside in the same unit cell volume. In fact, two monomers are assigned a contact probability γ only if they simultaneously occupy the same lattice site. This assumes that the dominant contributions to contacts between two chromosomal regions are from configurations where their respective centers occupy the same unit cell. Any excluded volume effects reducing the number of self-overlaps of the coarse-grained polymer manifest through imposed Hi-C score constraints.

To set the scale of the lattice spacing b in the model, we use the average spatial distance between consecutive Hi-C chromosomal regions determined in Note 2.1). If we consider distances between subsequent chromosomal regions, however, coarse-graining effects need to be taken into account: only seven distances between these regions are possible in the lattice representation (Supplementary Fig. 4 B): (0, $\sqrt{2}b$, $2b$, $\sqrt{6}b$, $\sqrt{8}b$, $\sqrt{10}b$, $4b$), which occur with respective relative occurrence frequencies ($f_1, \cdots, f_7$). In our MaxEnt model, we robustly observe ($f_1 \approx 0.092$, $f_2 \approx 0.50$, $f_3 \approx 0.13$, $f_4 \approx 0.19$, $f_5 \approx 0.041$, $f_6 \approx 0.048$, $f_7 \approx 0.0022$). This coarse-graining effect implies a cut-off of the tail of the underlying Gaussian distribution of 3D distances. To account for this cut-off, we first sample real-space configurations of consecutive chromosomal regions according to the experimentally determined 3D Gaussian distribution of continuous distances (see Note 2.1), and infer the statistics in the corresponding lattice model. For each of the seven possible (discretized) distances in the coarse-grained lattice representation, we thus obtain associated conditional distribution of real-space distances. The sum of the seven conditional real-space distance distributions, weighted by their respective relative occurrence frequencies (f_i), defines the full distribution of distances between neighbouring chromosomal regions in the MaxEnt model. We determine the lattice spacing $b = 88$ nm, such that the average distance between chromosomal regions in our MaxEnt model matches the experimentally determined average distance (Note 2.1)). Note, for this lattice spacing, the distribution of distances between neighbouring chromosomal regions in the MaxEnt model are also in accordance with our experimentally determined distributions (Supplementary Fig. 2).

The phase space of chromosome states is restricted to those that fit inside a cell, the sampling thus explores a constrained space (see also [9]). We introduce a confinement formed by a cylinder capped by two hemispheres. The dimensions of the confinement are chosen to match typical dimensions of a newborn swarmer cell. These dimensions are determined by taking a sample of 267 cells from the MvT151 data set, which yields an average length of $2.3 \pm 0.2, \mu m$ and width of $0.75 \pm 0.04 \mu m$, as determined by using the BacStalk software [10]. Subtracting the estimated width of the cell envelope of 61 nm (based on figure 2 of [11]), we arrive at typical chromosome confinement dimensions of $2.2 \times 0.63 \mu m$. With the inferred lattice spacing, this translates to a confinement of 470 unit cells (25 lattice spacings long and 7 wide). This representation of the cell could be refined further to include the crescent shape, but we find that such corrections do not appear to significantly affect the results of our model (see Note 17).

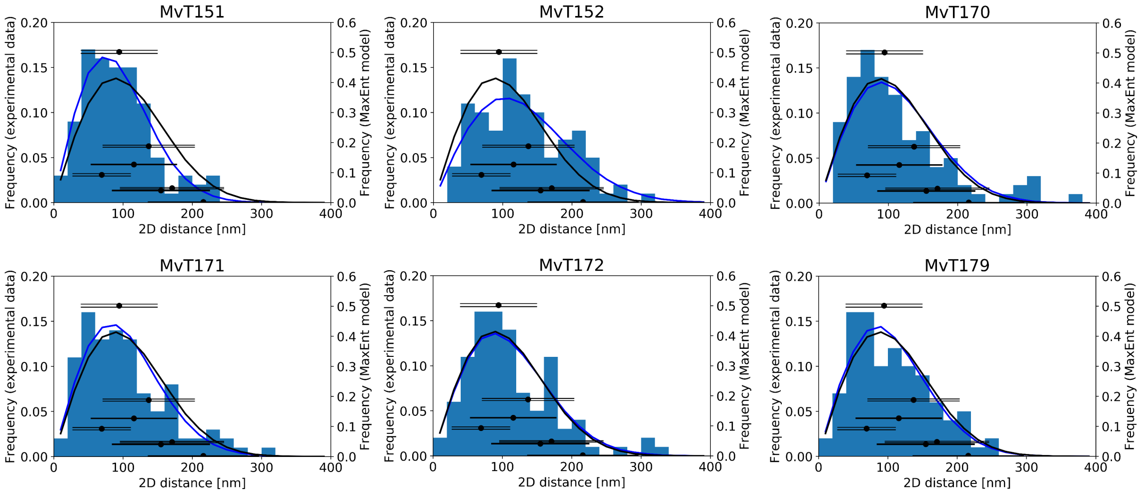

Supplementary Figure 2: Distributions of 2D projected distances from experiment and MaxEnt model. Bars: experimentally measured 2D distances (after bias correction, see Note 2.1). Blue lines: distributions of 2D projected distances from the inferred 3D Gaussian distribution. For each data set there is one fit parameter σ, chosen such that the average distances of measured and inferred distributions match. Black markers: Relative frequencies (f_i) of each of the seven possible configurations of two neighboring chromosomal regions of the MaxEnt model with associated average distances determined from coarse-graining. The pairs of horizontal black lines at each dot indicate the mean variance of the MaxEnt configuration frequency for all neighboring pairs of chromosomal regions. The error bar indicates the standard deviation of the underlying distance distribution for each coarse-grained configuration. Black curve: Inferred 2D distance distribution between consecutive genomic regions for the entire chromosome for the MaxEnt model. This distribution is obtained by weighing the inferred distance distribution for each coarse-grained configuration with the associated relative occupancy frequency within the MaxEnt model. To enable a direct comparison with experimental data, the inferred measurement noise is applied over the MaxEnt distance distribution. Note that all MaxEnt data sets are the same in each panel.

3 Inverse Monte Carlo algorithm for MaxEnt chromosome model

We solve the inverse problem and obtain the Lagrange multipliers ϵ_{ij}'s by an iterative procedure: we perform a Monte Carlo (MC) simulation (forward algorithm) to sample equilibrium states from the lattice polymer model with an initial guess for ϵ_{ij}. Subsequently, we compare the estimated contact map, f_{ij}^{sim}, obtained from this MC simulation, with the target experimental map f_{ij}^{expt}. When the modeled and experimental contacts deviate, the ϵ_{ij}'s are updated (inverse algorithm). This procedure converges when the modelled normalized contact frequency map matches the Hi-C data set within a tolerance level, yielding the complete set of parameters ϵ_{ij} that defines the MaxEnt model. The forward and inverse algorithm are described below.

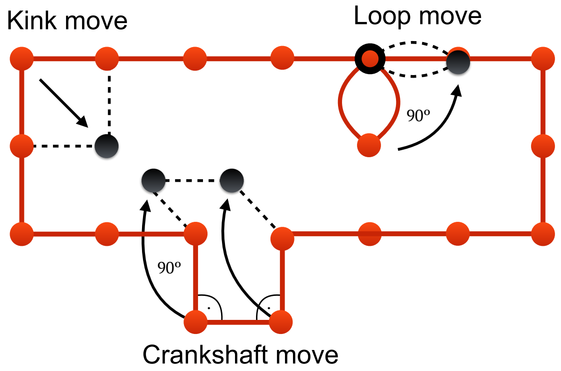

Supplementary Figure 3: Illustration of the three polymer moves employed in the Monte Carlo simulation. The simulation employs a kink move, a crankshaft move and a loop move.

3.1 Forward algorithm

In our coarse-grained model, the bacterial chromosome of *C. crescentus* is represented by a circular lattice polymer with a length of 1620 monomers. Each 4th monomer represents the location of the center of a genomic region, with three monomers in between to ensure Gaussian statistics between subsequent centers of genomic regions (see Note S2). The level of coarse-graining can be adapted to accommodate the resolution of the data on which the model is trained.

The algorithm is initiated with the circular polymer randomly arranged within the confinement. This starting state is obtained by first 'winding up' the polymer in a square that fits in the confinement. Subsequently, a simulation with no interaction energies is run for 10^7 Monte Carlo moves. The resulting configuration is used as the starting configuration. We simulate the Boltzmann distribution of polymer configurations in the MaxEnt model using Monte Carlo simulations. To sample configurations in the Monte Carlo algorithm, we employ three different polymer moves: the *kink move*, the *Crankshaft move* and the *loop move* (Supplementary Fig. 3). This move set preserves circularity and allows an ergodic sampling of the space of polymer configurations, which is demonstrated in Note 3.3. Moves which would place a monomer outside of the confinement are forbidden.

A potential move $\{\mathbf{r}\} \rightarrow \{\mathbf{r}'\}$ is randomly chosen (based on the move set in Supplementary Fig. 3), and then accepted with a probability $P_{\text{acc}}(\{\mathbf{r}'\}, \{\mathbf{r}\})$ according to the Metropolis criterion: $P_{\text{acc}}(\{\mathbf{r}'\}, \{\mathbf{r}\}) = \min(1, \exp(E(\{\mathbf{r}\}) - E(\{\mathbf{r}'\})))$, provided the configuration stays within the confinement. Here, $E(\{\mathbf{r}'\})$ and $E(\{\mathbf{r}\})$ are the energies of the proposed configuration $\{\mathbf{r}'\}$ and current configuration $\{\mathbf{r}\}$, respectively. The energies are computed according to the Hamiltonian (Eq. (5) in main text)

$$H(\{\mathbf{r}\}) = \frac{1}{2} \sum_{ij} \epsilon_{ij} \delta_{\mathbf{r}_i, \mathbf{r}_j}. \tag{S1}$$

For pairs of genomic regions i, j for which $\tilde{f}_{ij}^{\text{expt}} = 0$, the corresponding ϵ_{ij} is set to a high value at the start of the simulation, typically 10, which may further increase during iterations of the inverse algorithm. Note, this initial value is high enough to ensure these contacts do not form in practice. At the start of the forward simulation, we apply a burn in time of 2×10^7 MC moves before contact

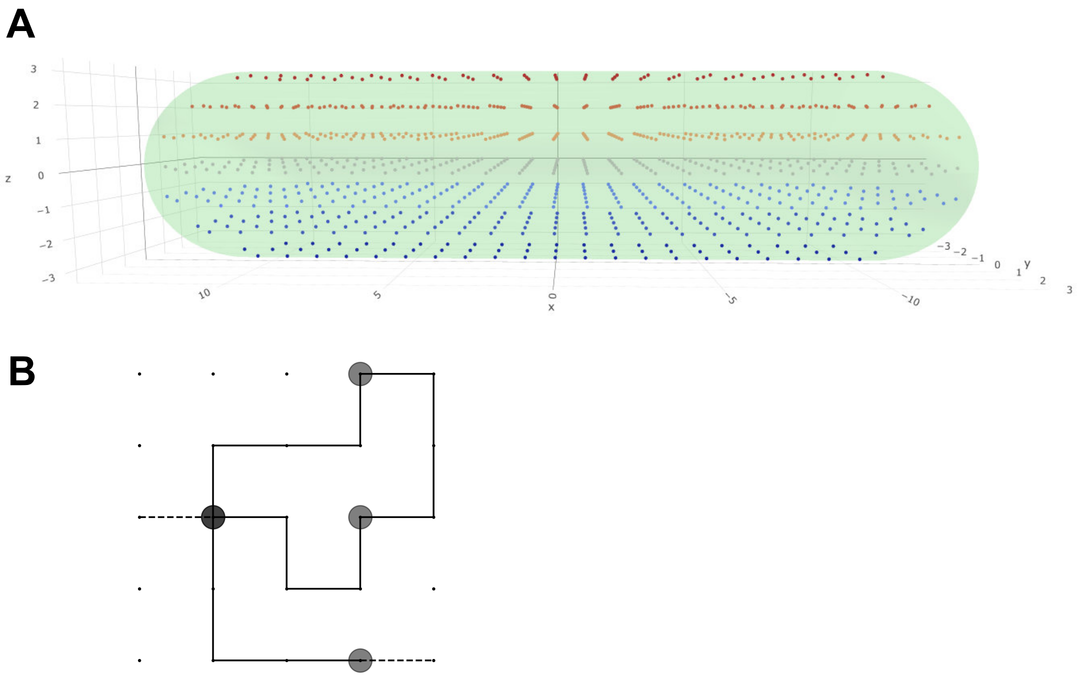

Supplementary Figure 4: Illustration of the model confinement and chromosome representation A The cellular confinement used in the simulations. Each dot represents a lattice point. **B** Illustration of the coarse-grained representation of the chromosome, which is shown here in 2D for simplicity. The chromosome is represented by a lattice polymer, where each fourth monomer describes the position of the center of a genomic region. The three monomers in between centers of genomic regions serve to ensure correct distance statistics between subsequent genomic regions. When two centers of genomic regions overlap, they have a probability γ of forming a contact that contributes to the Hi-C map.

frequency statistics are calculated. During the inverse algorithm, this burn in time is only applied to the first forward simulation. For subsequent forward simulations, the final configuration of the previous forward simulation is used as a starting state.

3.2 Inverse algorithm

As noted in the main text, we learn the MaxEnt model directly from the normalized experimental Hi-C map. During a forward simulation of the polymer, the contact frequency f_{ij}^{model} of each pair of monomers is counted. After one round of forward simulation, the simulated contact frequencies are normalized and compared to the experimental ones. The pairwise interaction energies are then updated according to

$$\Delta\epsilon_{ij} = \alpha(\tilde{f}_{ij}^{\text{model}} - \tilde{f}_{ij}^{\text{exp}}) \times \frac{1}{\sqrt{\tilde{f}_{ij}^{\text{exp}}}}. \tag{S2}$$

Here, α is the learning rate (which we typically set to 0.2), and the last factor is included to speed up conversion for pairs with a low contact frequency. Note, $\tilde{f}_{ij}^{\text{model}}$ and $\tilde{f}_{ij}^{\text{exp}}$ are the normalized model and experimental contact frequencies, respectively.

Importantly, to impose that the normalized contact frequencies match between model and experiment, we need to determine one remaining parameter: the absolute scale of the model contact frequencies. This is fixed by main text Eq. 6, which is derived as follows. Writing $f_{ij}^{\text{expt}} = c\tilde{f}_{ij}^{\text{expt}}$ and $\tilde{c} = \frac{c}{\gamma}$, the entropy functional becomes

$$\tilde{S} = -\sum_{\{\mathbf{r}\}} P(\{\mathbf{r}\}) \ln P(\{\mathbf{r}\}) - \sum_{ij} \lambda_{ij} \Bigg(\sum_{\{\mathbf{r}\}} P(\{\mathbf{r}\}) \delta_{\mathbf{r}_i, \mathbf{r}_j} - \tilde{c}\tilde{f}_{ij}^{\text{expt}} \Bigg) - \lambda_0 \Bigg(\sum_{\{\mathbf{r}\}} P(\{\mathbf{r}\}) - 1 \Bigg) \tag{S3}$$

we require that $\tilde{c}$ maximizes the model entropy, setting $\frac{\delta \tilde{S}}{\delta \tilde{c}} = 0$. This yields main text Eq. 6:

$$\sum_{ij} \lambda_{ij} \tilde{f}_{ij}^{\text{expt}} = 0. \tag{S4}$$

Ensuring that this condition is satisfied in each iteration step fixes the overall scale of contact frequencies. In the simulation, this is done by applying an overall shift in the interaction energies after the update step in Eq. (S2). This overall shift can be derived as follows: we start from Eq. 6, which imposes $\sum_{ij} \epsilon_{ij} \tilde{f}_{ij}^{\text{expt}} = 0$. In general, a set of ϵ_{ij} obtained after the update step in Eq. (S2) will not satisfy this constraint. We can, however, introduce a shift $\Delta\epsilon$ of all ϵ_{ij} such that this condition is satisfied:

$$\sum_{ij} (\epsilon'_{ij} - \Delta\epsilon) \tilde{f}_{ij}^{\text{expt}} = 0. \tag{S5}$$

Rewriting, and making use of $\sum_{ij} \tilde{f}_{ij}^{\text{expt}} = N_{\text{bin}}$ with N_{bin} is the number of Hi-C bins, yields

$$\Delta\epsilon = -\frac{\sum_{kl} \epsilon'_{kl} f_{kl}^{\text{exp}}}{N_{\text{bin}}}. \tag{S6}$$

Performing this shift after each update step ensures that the condition in main text Eq. 6 is satisfied at each iteration of the inverse algorithm.

We iterate the inverse algorithm until the Pearson's correlation coefficient between the simulated normalized contact frequencies and the experimental data is above 0.98. This is the correlation coefficient of contact frequencies between repeat experiments reported in [12]. In practice, we can obtain even higher correlation coefficients of 0.998, as stated in the main text. With each subsequent forward simulation, the number of Monte Carlo steps is multiplied by $\sqrt{n}$, with n the iteration step. The inverse algorithm is typically started with $\sim$ 360 million steps, and run for $\sim$ 100 iterations.

3.3 Ergodicity of forward algorithm

Next, we demonstrate that the algorithm is ergodic. A circular path of the polymer can be represented as a sequence of N steps along the lattice, where each step is either up (U), down ($\bar{U}$), right (R), left ($\bar{R}$), in (I) or out ($\bar{I}$). We denote the total number of steps of type x by $N(x)$. Circularity

of the path implies that $N(U) = N(\bar{U})$, $N(R) = N(\bar{R})$ and $N(I) = N(\bar{I})$. Furthermore, we will divide the steps in *types*, where (U) and $(\bar{U})$ are type 1, (R) and $(\bar{R})$ are type 2, and (I) and $(\bar{I})$ are type 3. An individual path can then be described as a sequence of steps, for example

$$[\mathrm{U}, \bar{R}, \mathrm{I}, \mathrm{R}, \bar{U}, \cdots]. \tag{S7}$$

Here, each of the steps is colored by type. In the following we will also consider the sequence within each type. For our example, the sequences for the three types are:

- Type 1: $[\mathrm{U}, \bar{U}, \cdots]$
- Type 2: $[\bar{R}, \mathrm{R}, \cdots]$
- Type 3: $[\mathrm{I}, \cdots]$

We now consider the action of each of the polymer moves on a sequence of steps.

- The **kink move** interchanges two subsequent steps of a different type. Using only this move, any sequence of type 1, type 2 and type 3 steps can be created from a starting sequence that doesn't change the number of each type. Put differently, using the representation in (S7), any sequence of red, green and blue can be created that conserves the original counts of each color. Within each type, the sequence of the possible steps (e.g. U and $\bar{U}$), however, cannot be changed with this move.

- The **crankshaft move** takes a motif of the form $[A, B, \bar{A}]$ and alters this to one of three possible motifs: (i) $[\bar{A}, B, A]$, or (ii) $[C, B, \bar{C}]$, or (iii) $[\bar{C}, B, C]$. The first alteration changes the sequence of steps within a type. Combining this alteration with the kink move, any sequence of steps within each type can be created, provided that there is at least one set of steps of a different type.

  Alteration (ii) and (iii) change the number of steps of each type: $N(A) + N(\bar{A})$ is reduced by 2, and $N(C) + N(\bar{C})$ is increased by 2. Combining this with the kink move, any set of counts of each of the types can be created, provided that polymer length and circularity are preserved, and that in the initial state not all steps are of the same type.

  Combining all three alterations with the kink move, from any starting sequence any final sequence can be created that conserves polymer length and circularity, as long as the starting and final sequence have moves of at least two different types.

- The **loop move** takes a motif of the form $[A, \bar{A}]$ and alters it to either (i) $[\bar{A}, A]$ or (ii)$[B, \bar{B}]$ or (iii) $[\bar{B}, B]$. Alteration (i) enables any change of the sequence within a type when the entire initial sequence is of the same type. Alterations (ii) and (iii) allow the conversion from a state of only one type to a state of two types.

  Combining the loop move with the kink and crankshaft moves, from any starting sequence any final sequence can be created that conserves polymer length and circularity. Thus, an ergodic sampling of the space of polymer configurations is ensured.

Note I: The presence of a confinement introduces a parity on the lattice sites: sites that can be occupied by an even monomer through these 3 moves cannot be occupied by an uneven monomer,

and vice versa. Either choice of parity can be seen as a separate coarse-grained model, as the unit cell locations shift depending on this choice.

Note II: A confinement could be chosen that 'traps' a portion of the polymer in place, making the phase space reachable using the three moves dependent on the initial state. For our confinement consisting of a cylinder with rounded edges such a trapping is not present, thus ergodicity is still preserved.

Note III: ergodicity is already ensured if only the loop and kink moves are used: the crankshaft move can be constructed as a combination of the two. However, the crankshaft move allows for a faster exploration of phase space and is thus also included.

4 Testing the inverse Monte Carlo algorithm

To test the performance of our inverse algorithm, we generated trial data sets by running a forward simulation for a chosen set of input effective interaction energies $\epsilon_{ij}^{\text{in}}$ (upper left Supplementary Fig. 5A). The resulting simulated contact map, f_{ij}^{in}, exhibits intricate features, including domain-like structures along the main diagonal and a fainter second diagonal (upper left Supplementary Fig. 5B). Subsequently, we treat this contact map as an experimental data set, which we use as an input to our iterative inverse scheme. We find that our inverse scheme rapidly and accurately retrieves the correct energies, $\epsilon_{ij}^{\text{model}} \approx \epsilon_{ij}^{\text{in}}$, and contact frequencies, $f_{ij}^{\text{model}} \approx f_{ij}^{\text{in}}$, demonstrating that this scheme adequately solves the inverse problem (Supplementary Fig. 5A-C).

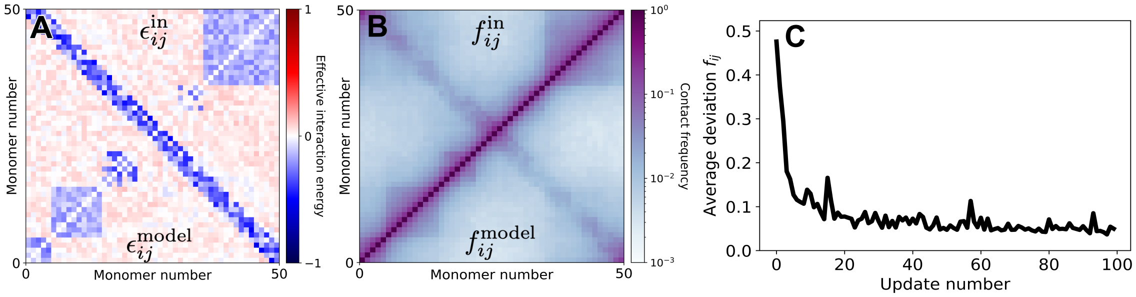

Supplementary Figure 5: Demonstration of numerical inverse algorithm for MaxEnt chromosome model. A Upper left: input effective interaction energies $\epsilon_{ij}^{\text{in}}$. Lower right: effective interaction energies retrieved by the MaxEnt model. **B** Upper left: simulated contact frequencies f_{ij}^{in} using $\epsilon_{ij}^{\text{in}}$. Lower right: contact frequencies of the MaxEnt model, using f_{ij}^{in} as an input. **C** The average relative contact frequency deviation: $\langle f_{ij}^{\text{in}} - f_{ij}^{\text{model}} \rangle / \langle f_{ij}^{\text{model}} \rangle$ vs. iteration number of inverse algorithm.

5 Hi-C data filtering

Before the Hi-C data from Ref. [12] can be used to train our single-chromosome MaxEnt model, we need to account for the presence of a small fraction of replicating cells due to imperfect synchronization. Most notably, there is a local increase in Hi-C scores between the *ori* and *ter* genomic

regions, which is attributed to a small fraction of cells that have partially replicated their chromosome and segregated their newly formed *ori* regions towards the other pole, where the *ter* region of the initial chromosome is located [13, 14]. Although this increase is not readily visible on a linearly scaled Hi-C map (Supplementary Fig. 6A, upper left), it is clearly visible on a logarithmic scale (Supplementary Fig. 6A, lower right). Importantly, in experiments where replication is inhibited prior to synchronization, such an increase in contacts between the *ori* and *ter* genomic regions is not observed [15] (Supplementary Fig. 6B). In Ref. [15], two Hi-C experiments were performed on swarmer cells that could not undergo replication or cell division: on cells depleted of *dnaA*, and cells overexpressing *ctrA(D51E)*$\Delta 3\Omega$.

5.1 DNA replication inhibited Hi-C datasets

In the cells depleted of DnaA, the only copy of *dnaA*, whose product activates the initiation of replication, is driven by an IPTG-regulated promoter. Growth in medium lacking IPTG produced a population of cells that contained only a single, unreplicated copy of the chromosome. Cells were suspended in PYE medium without IPTG to deplete DnaA for 90 min before synchronization [15]. The data set analyzed here is for cells that were formadehyde fixed immediately after synchronization (90 min after IPTG withdrawal).

In the cells overexpressing the hyperactive and non-degradable CtrA variant *ctrA(D51E)*$\Delta 3\Omega$, chromosome replication is inhibited by constitutive binding of CtrA close to the origin of replication. *ctrA(D51E)*$\Delta 3\Omega$ is expressed from an xylose-inducible promoter on the high copy number pJS14 plasmid in the presence of the chromosomal copy of wild-type *ctrA*. Cells were suspended in PYE medium plus xylose for 60 min before synchronization [15]. The data set analyzed here is for cells that were formadehyde fixed at 0 hr post synchronization (60 min after xylose addition).

For both the DnaA-depleted cells and the cells overexpressing *ctrA(D51E)*$\Delta 3\Omega$, average Hi-C scores are found to monotonicaly decrease with inter-arm genomic distance until a noise floor is reached, and to exhibit three distinct scaling regimes (Supplementary Fig. 6C). By contrast, for the wild-type synchronized swarmer cells from Ref. [12], an increase in average Hi-C scores for the largest inter-arm genomic distances is observed (Supplementary Fig. 6F). If we train a MaxEnt model directly on this data, this single-chromosome model will interpet these *ori-ter* contacts as inter-chromosomal contacts, resulting in a weaker localization of the *ter* region (Supplementary Note 7). Here, we propose a filtering procedure to process the wild-type data such that we can infer a reliable single-chromosome MaxEnt model, even in the presence of a small fraction of non-synchronized cells.

5.2 Filter procedure

The goal of our data processing procedure is to filter out the contribution of the newly replicated *ori* from the wild-type data set, using the two data sets for replication-inhibited cells as a benchmark. The advantages of filtering the wild-type dataset, rather than applying the analysis to the replication-inhibited cells, are two-fold: First, the experimental procedure to inhibit replication might affect features of chromosome organization. Second, a filter method allows for the analysis of data sets for mutants and cells in atypical growth conditions but without replication inhibition, such as the Δ*smc* mutant and the rifampicin-treated cells in Ref. [12], using a single chromosome model. For completeness, the results of applying the MaxEnt method directly to the unfiltered wild-type data, as well as to the replication-inhibited cell data, are presented in Supplementary

Notes 7 and 8. Importantly, we find that all the central conclusions drawn in the Main Text based on our MaxEnt model trained on the filtered WT data, can also be drawn for a MaxEnt model on the unprocessed Hi-C data from the replication-inhibited cells.

The procedure to filter out the contribution of the newly replicated *ori* aims to reproduce three features observed for replication-inhibited cells: (1) a power law scaling of the average contact frequencies in regime III (Supplementary Fig. 6C), (2) a proportionality between the mean and variance of Hi-C scores across inter-arm genomic distance bins (Supplementary Fig. 6D), and (3) a transition to a noise floor regime for the largest inter-arm genomic distances (Supplementary Fig. 6C,E). The filtering procedure is as follows. First, the estimated average Hi-C scores for the single, unreplicated chromosome $f_{\rm av}^{\rm single}(d)$ are constructed for each inter-arm genomic distance bin, d, in regime III (Supplementary Fig. 6F, red dashed line) for the wild-type data set (the construction procedure is detailed in the next paragraph). A rescaling factor $\mu(d) = \frac{f_{\rm av}^{\rm single}(d)}{\langle f_{ij}^{\rm WT}\rangle_d}$ is then obtained between $f_{\rm av}^{\rm single}(d)$ and the unfiltered wild-type data averages $\langle f_{ij}^{\rm WT}\rangle_d$ for a given distance bin d. This factor $\mu(d)$ is subsequently used to rescale individual Hi-C scores of the wild type data set at each inter-arm genomic distance bin d within regime III. By construction, this rescaling procedure ensures that the filtered Hi-C scores will not only have the correct estimated average value, but also the correct estimated variance, preserving the proportionality between average Hi-C scores and the asscociated variance observed for replication-inhibited cells. Finally, when the average rescaled contact frequencies fall below the noise floor observed for replication-inhibited cells, Hi-C scores are determined from the observed noise floor distribution (Supplementary Fig. 6E).

To construct $f_{\rm av}^{\rm single}(d)$, we need two points a and b on the log-log plot to define the power law relation associated to regime III. The vertical position of point a is set at $\langle f_{ij}^{\rm WT}\rangle_d$ at the onset of regime III (Supplementary Fig. 6F), beyond which contributions from the newly replicated *ori* are assumed to become significant. To position point b, we assume the contributions to $\langle f_{ij}^{\rm WT}\rangle_d$ from inter-chromosomal contacts and the newly replicated *ori* regions to be equal at the minimum of $\langle f_{ij}^{\rm WT}\rangle_d$ (Supplementary Fig. 6F, dash-dotted line), since this marks the distance beyond which contributions from the newly replicated *ori* become dominant. We thus set the vertical position of point b equal to $\langle f_{ij}^{\rm WT}\rangle_d/2$. Hence, $f_{\rm av}^{\rm single}(d)$ follows the power law relation consistent with the line from a to b, extending till point c, where the noise floor level is reached; this noise floor is found to be at an average Hi-C score of 0.000078 for the replication-inhibited cells(Supplementary Fig. 6F, point c). Using this procedure, we now also obtain $\mu(d)$ between point a and c.

The filter procedure rescales the Hi-C data by $\mu(d)$ between points a and c in regime III. Beyond point c, the noise level is reached, and Hi-C scores are randomly drawn from the observed noise floor distribution. These noise-floor distributions are constructed by counting all Hi-C scores of the two replication-inhibited cells with an inter-arm genomic distance above 1.78 Mb, and are consistent with an underlying Poissonian process (Supplementary Fig. 6E). Importantly, this construction leaves all Hi-C scores in scaling regimes I and II (Supplementary Fig. 6C) unchanged, and filters wild-type Hi-C scores in regime III. The resulting filtered Hi-C scores are shown in Supplementary Fig. 6G and Supplementary Fig. 6H. Finally, we applied the same data processing procedure to two other replicas of the WT experiments, as shown in Supplementary Fig. 7.

Our data processing procedure ensures that the averages and variances of the contact frequencies per inter-arm genomic distance bin behave as observed for replication-inhibited cells. However, it is possible that additional structure is present in the replication-inhibited data, which is lost in the filtered wild-type data during this data processing procedure. To test this, we compute the correlation between Hi-C contact scores within each inter-arm genomic distance bin, between (1)

the filtered wild-type data set, (2) the DnaA-depleted cells and (3) *ctrA(D51E)*$\Delta 3\Omega$ overexpressing cells. These correlations are a measure for the similarities between the data sets for each inter-arm genomic distance bin. A correlation of 1 corresponds to two data sets being identical up to a proportionality constant, whereas a correlation of 0 corresponds to the variations within a genomic distance bin being linearly independent between data sets. At the onset of regime III (point a), we find significant correlations between the three datasets (Supplementary Fig. 6I). Importantly, these correlations do not significantly differ between each of the three pairs of data sets, indicating the presence of similar structure in the filtered wild-type data set and the replication-inhibited data sets. For larger inter-arm genomic distances, the correlations between data sets go to zero, as would be expected at the onset of the noise floor regime (point c).

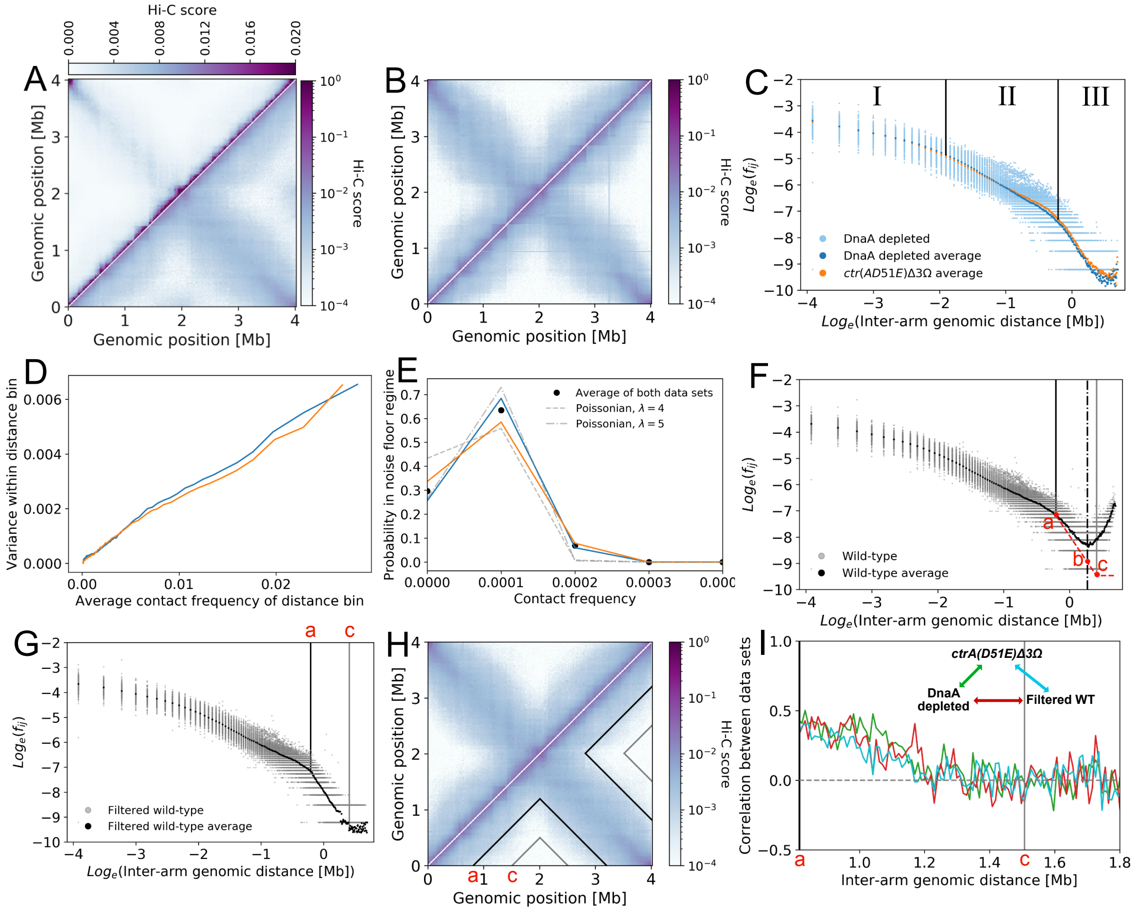

Supplementary Figure 6 ***(previous page)*****: Hi-C data processing procedure A** Wild-type contact frequencies from [12] on a linear scale (upper left triangle) and a logarithmic scale (bottom right triangle). **B** Contact frequencies for replication-inhibited swarmer cells directly after synchronization. Upper left triangle: DnaA-depleted cells; lower right triangle: cells overexpressing *ctrA(D51E)*$\Delta 3\Omega$. Both datasets are taken from [15]. **C** Hi-C score versus inter-arm genomic distance for DnaA depleted cells (light blue dots). Dark blue dots: averages per inter-arm genomic distance bin. Orange dots: averages per inter-arm genomic distance bin for cells overexpressing *ctrA(D51E)*$\Delta 3\Omega$. Three distinct scaling regimes are identified, indicated by regions I-III. **D** Variance of Hi-C scores within an inter-arm genomic distance bin versus the average Hi-C score of this genomic distance bin for DnaA-depleted cells (blue line) and the cells overexpressing *ctrA(D51E)*$\Delta 3\Omega$ (orange line). **E** Probabilities of Hi-C score occurances in the noise floor regime, taken over pairs of genomic regions with an inter-arm genomic distance of at least 1.78 Mb. Blue line: DnaA-depleted cells; orange line: cells overexpressing *ctrA(D51E)*$\Delta 3\Omega$; black dots: averages per Hi-C score bin; dashed line: distribution for a poissonian process with a mean equal to the average of the two data sets and $\lambda = 4$; dash-dotted line: the same for $\lambda = 5$. **F** Hi-C score versus inter-arm genomic distance for wild-type cells (grey dots). Black dots: average per inter-arm genomic distance bin. Dash-dotted line: horizintally aligned with the minimum point of the average Hi-C scores. Red dashed line: $f_{\rm av}^{\rm single}(d)$ (from point a to c) and the noise floor beyond point c (see Supplementary text for more details). **G** Wild-type Hi-C scores after the filtering procedure is applied (grey dots) together with the averages per inter-arm genomic distance bin (black dots). **H** Wild-type Hi-C score map after the filtering procedure has been applied. Hi-C scores have been rescaled in the regime between the black and the grey lines. The noise floor region is enclosed withn the grey lines, where Hi-C scores have been randomly drawn from the distribution in **E**. **I** Correlations of contacts within an inter-arm genomic distance bin, between (1) the filtered wild-type data set, (2) the DnaA-depleted cells and (3) *ctrA(D51E)*$\Delta 3\Omega$ overexpressing cells.

6 Comparison of filter procedure for wild-type replicates

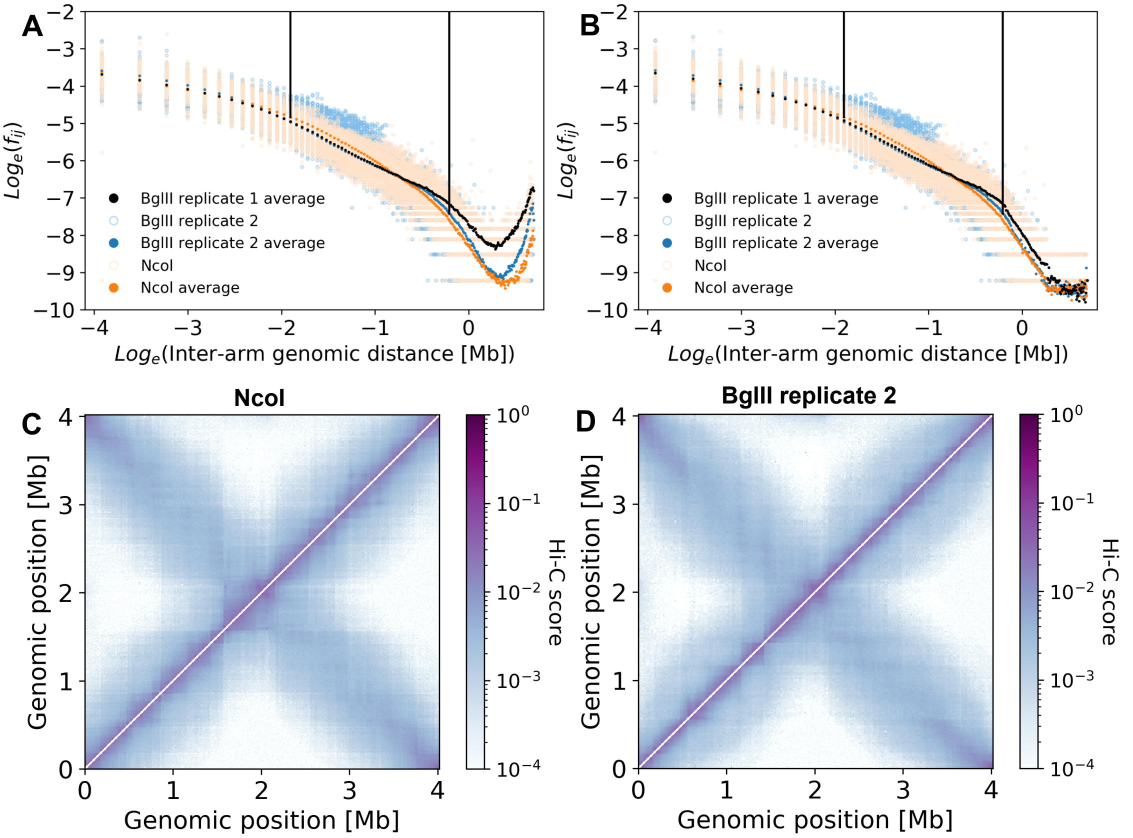

Supplementary Figure 7: Comparison of data filtering procedure for wild-type replicates A Unfiltered Hi-C scores as a function of inter-arm genomic distance for the three wild-type replicates published in [12]. The onsets of scaling regimes II and III as introduced in Supplementary Notes 5 are indicated by black vertical lines. **B** Hi-C scores versus inter-arm genomic distance for the three replicates after the filter procedure has been applied. **C** Upper left: Hi-C scores of the NcoI dataset before the filter procedure is applied. Lower right: Hi-C scores of the same data set after the filter procedure has been applied. **D** Upper left: Hi-C scores of the BglII replicate 2 dataset before the filter procedure is applied. Lower right: Hi-C scores of the same data set after the filter procedure has been applied.

7 Results for MaxEnt model trained on unfiltered Hi-C data

To further investigate the effect of this data processing on the model results, we reran our analysis directly on the unprocessed Hi-C data. We find that the localizations of genomic regions (Supplementary Fig. 9), the orientational and radial correlations in positions of regions (Supplementary Fig. 10) and the local structure (Supplementary Fig. 11) are largely unaffected. The most significant difference is found in the localization of the *ter* region, which is now found to move throughout the

ori half of the cell in a minority of states (Supplementary Fig. 9). This movement of the terminus has an effect on the long-axis anti-correlations between the *ori* and *ter* regions (Supplementary Fig. 10), resulting in a modified long-axis correlation pattern compared to the filtered Hi-C data (Main Text Fig. 3B). However, if conditional long-axis correlations are computed, conditioned on the *ori* region (here defined as 3.75 Mb - 0.25 Mb) being in one half of the cell, and the *ter* region (here defined as 1.75 Mb - 2.25 Mb) being in the other half, the pattern of anti-correlations between the two juxtaposed chromosomal arms is restored (Supplementary Fig. 10).

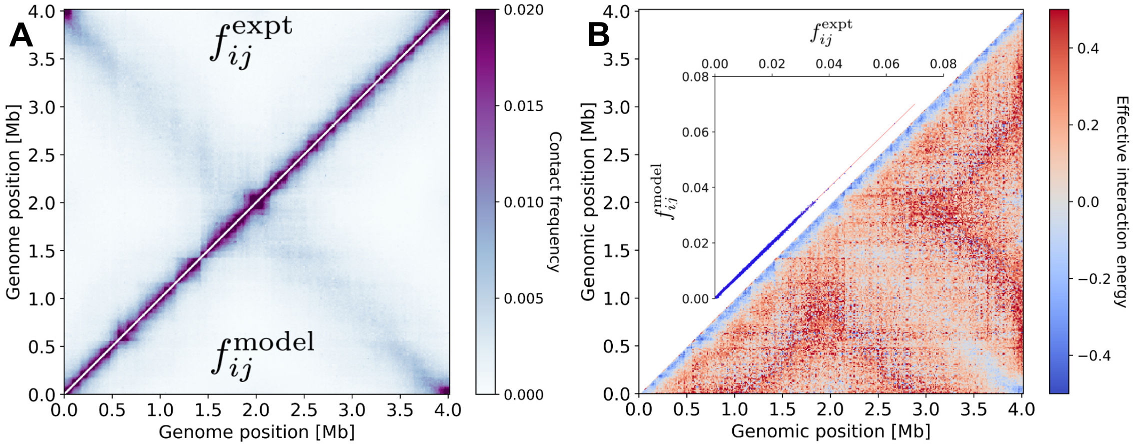

Supplementary Figure 8: Results for Main Text Fig. 1, re-analyzed for the unfiltered Hi-C data of replicate 1 from [12]. A Comparison between experimental contact frequencies f_{ij}^{expt} (upper left corner, adapted from Ref. [12]) and contact frequencies obtained from our inferred MaxEnt model f_{ij}^{model} (lower right corner). **B** Associated inferred effective interaction energies ϵ_{ij} (lower right corner, white regions indicate $\epsilon_{ij} \to \infty$) together with a scatter plot of f_{ij}^{expt} vs. f_{ij}^{model} (inset).

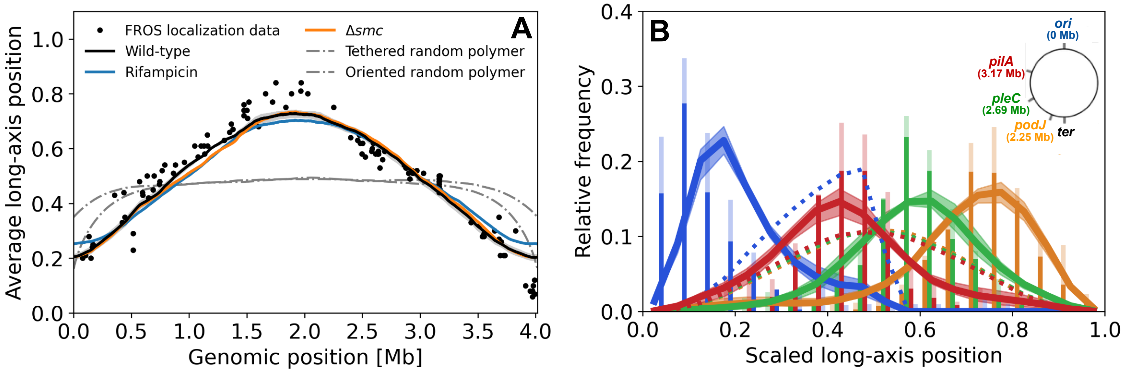

Supplementary Figure 9: Results for Main Text Fig. 2, re-analyzed for the three replicates from [12]. **A** Black solid line: average of the three data sets. Grey area: standard deviation of the three replicates, centered at the average. **B** Solid lines: averages of the three replicates. Shaded areas: standard deviations of the three replicates, centered at the average. Bars: experimental data from microscopy experiments (adapted from [3]). To indicate experimental variability, the solid/transparent bars indicate the minimum/maximum measured by two different methods: FROS or FISH.

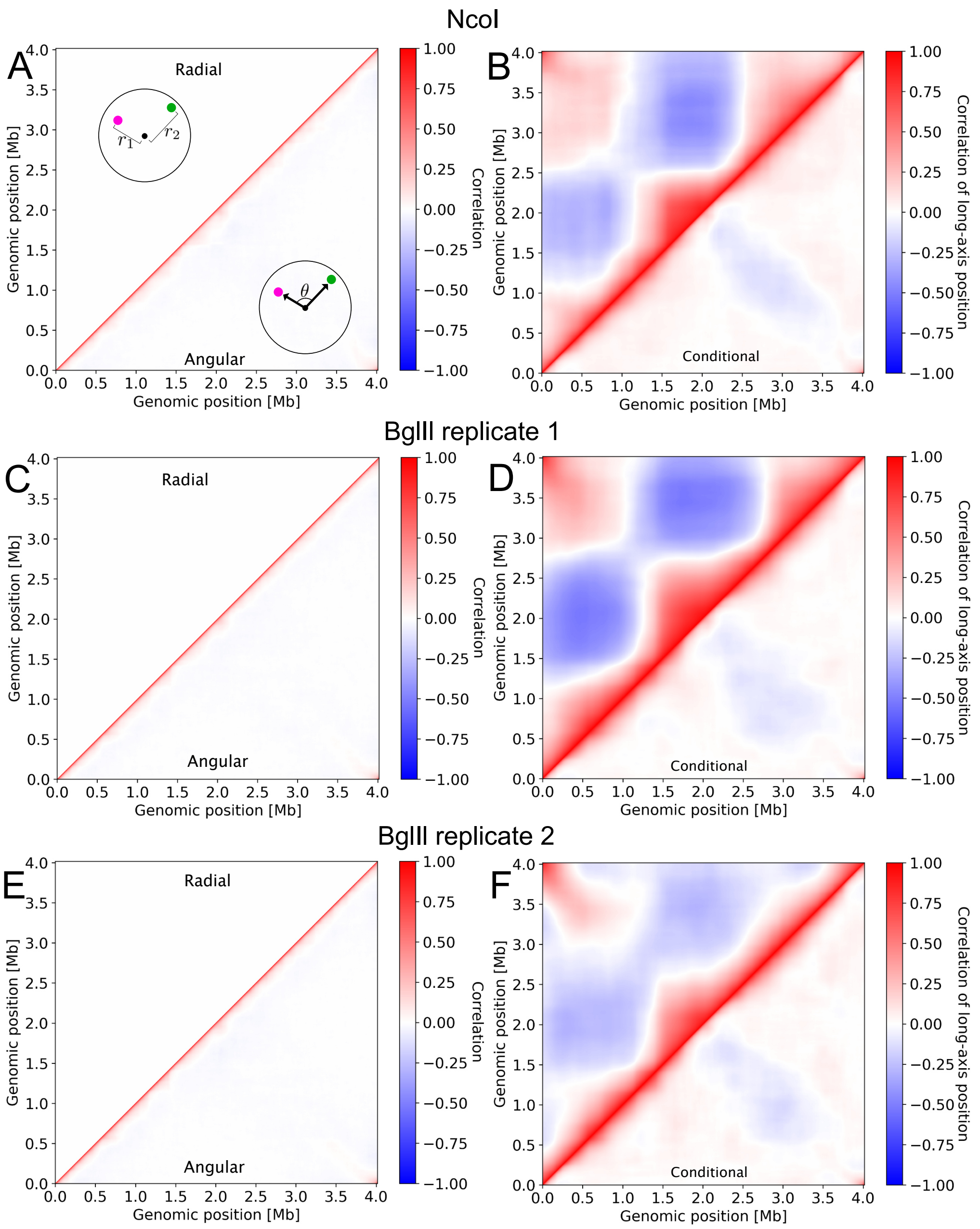

Supplementary Figure 10: Two-point correlations for the three replicates from [12]. Plots **A** and **B** are for the NcoI replicate. **A** Upper left corner: two-point correlations in the radial positions between genomic regions. Lower right corner: two-point correlations in angular orientations around the long axis. **B** Upper left corner: two-point correlations between long-axis positions of genomic regions. Lower right corner: conditional long-axis correlations, conditioned on the *ori* region (here defined as 3.75 Mb - 0.25 Mb) being in one half of the cell, and the *ter* region (here defined as 1.75 Mb - 2.25 Mb) being in the other half. **C** and **D**: same as **A** and **B**, for BglII replicate 1. **E** and **F**: same as **A** and **B**, for BglII replicate 2.

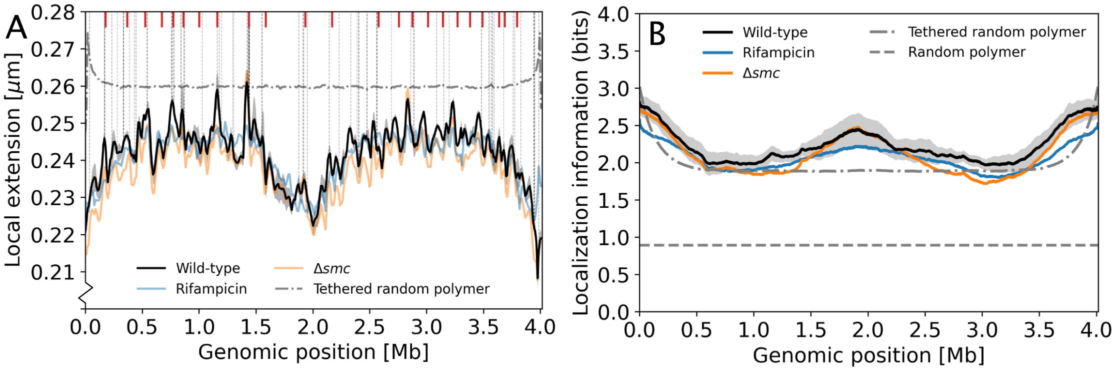

Supplementary Figure 11: Results for Main Text Fig. 5, re-analyzed for the three replicates from [12]. **A** The local chromosome extension δ_i as a function of genomic position. Black solid line: average of the three replicates. Grey areas: standard deviation of the three replicates, centred at the average. **B** Localization information per genomic region in bits. Black solid line: average of the three replicates. Grey areas: standard deviation of the three replicates, centred at the average.

8 Results for MaxEnt model trained on replication-inhibited cells

8.1 DnaA-depleted cells

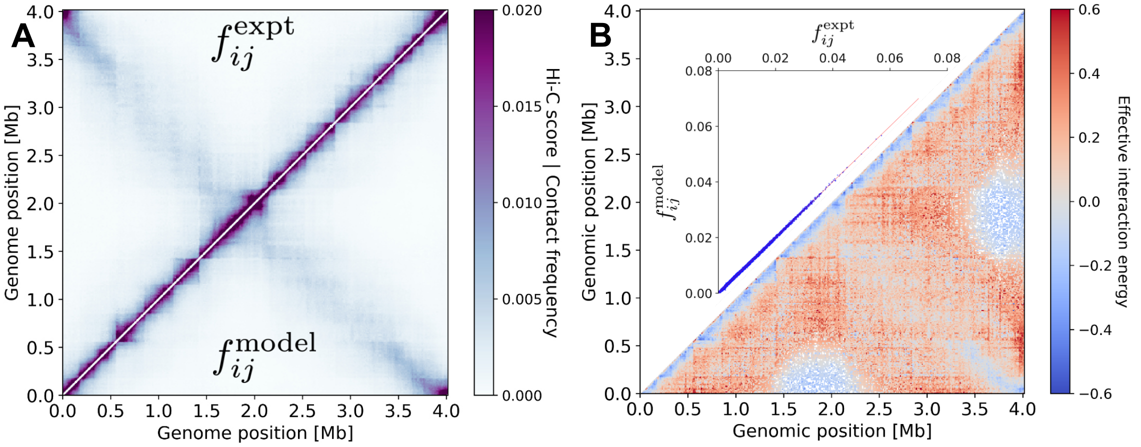

Supplementary Figure 12: Results for Main Text Fig. 1, re-analyzed for the DnaA-depleted cell data set from [15]. A Comparison between experimental contact frequencies f_{ij}^{expt} (upper left corner, adapted from Ref. [15] and contact frequencies obtained from our inferred MaxEnt model f_{ij}^{model} (lower right corner). **B** Associated inferred effective interaction energies ϵ_{ij} (lower right corner, white regions indicate $\epsilon_{ij} \to \infty$) together with a scatter plot of f_{ij}^{expt} vs. f_{ij}^{model} (inset).

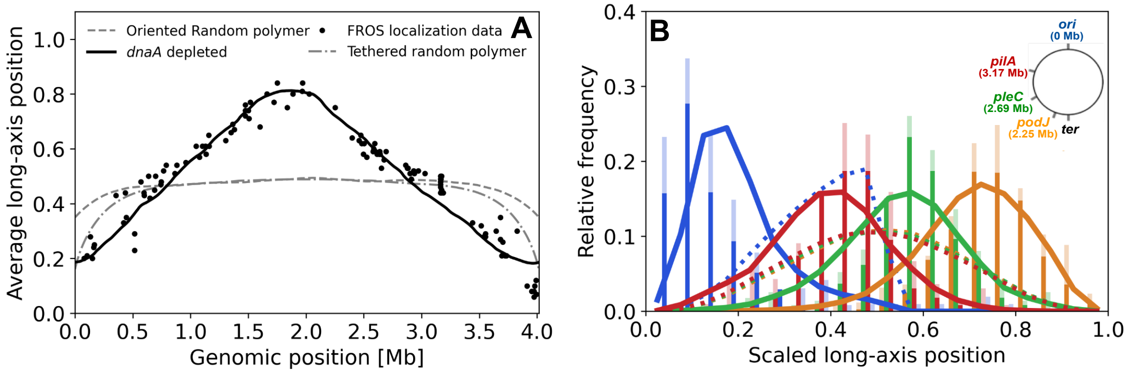

Supplementary Figure 13: Results for Main Text Fig. 2, re-analyzed for the DnaA-depleted cell data set from [15]. **A** Black solid line: average long-axis positions of genomic regions for DnaA-depleted cells predicted by the MaxEnt model. **B** Solid lines: distribution of long-axis positions of chromosomal loci (blue: *ori*, red: *pilA*, green: *pleC*, orange: *podJ*) for DnaA-depleted cells predicted by the MaxEnt model, , together with previous experimental data from microscopy experiments (bars, adapted from [3]). To indicate experimental variability, the solid/transparent bars indicate the minimum/maximum measured by two different methods: FROS or FISH.

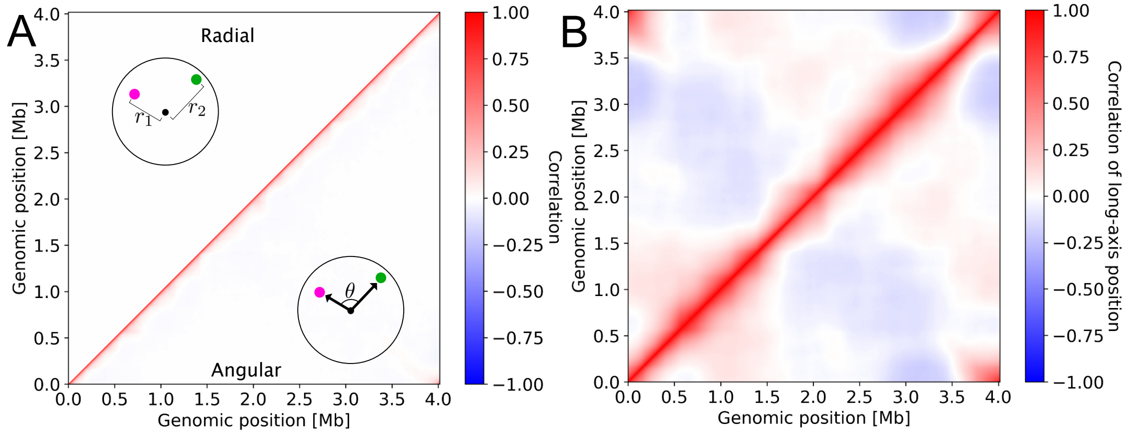

Supplementary Figure 14: Results for Main Text Fig. 3, re-analyzed for the DnaA-depleted cell data set from [15]. **A** Upper left corner: two-point correlations in the radial positions between genomic regions. Lower right corner: two-point correlations in angular orientations around the long axis. **B** Two-point correlations between long-axis positions of genomic regions.

8.2 Cells overexpressing *ctrA(D51E)*$\Delta 3\Omega$

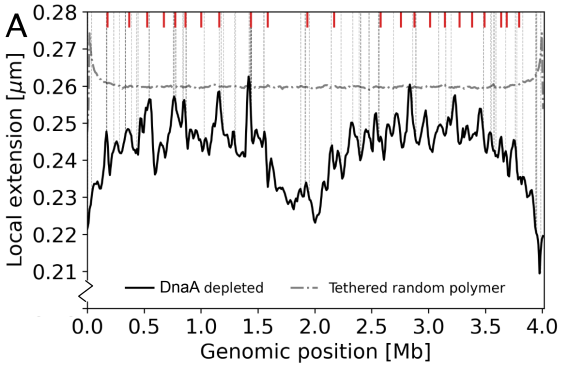

Supplementary Figure 15: Results for Main Text Fig. 5, re-analyzed for the DnaA-depleted cell data set from [15]. A Black solid line: the local chromosome extension δ_i as a function of genomic position for DnaA-depleted cells as predicted by the MaxEnt model. **B** Black solid line: localization information per genomic region for DnaA-depleted cells as predicted by the MaxEnt model.

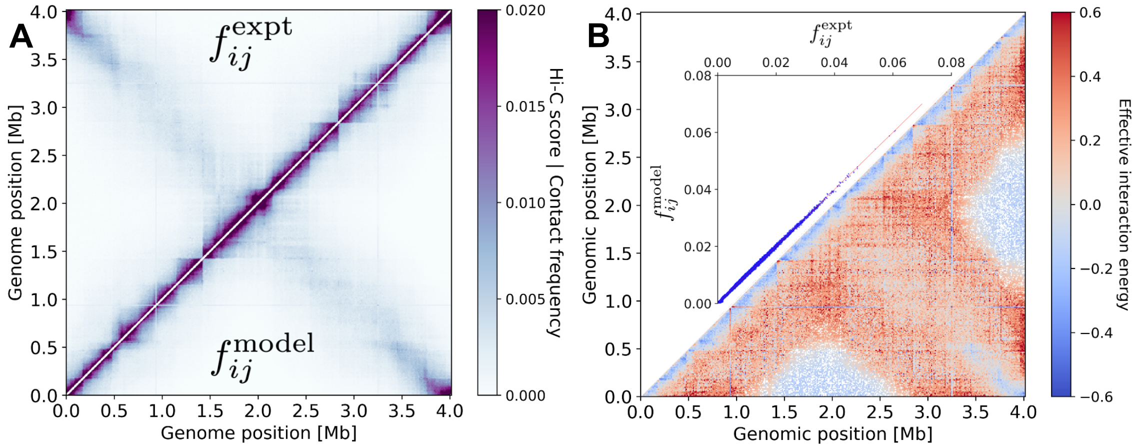

Supplementary Figure 16: Results for Main Text Fig. 1, re-analyzed for the *ctrA(D51E)Δ3Ω* overexpressing cell data set from [15]. A Comparison between experimental contact frequencies f_{ij}^{expt} (upper left corner, adapted from Ref. [15] and contact frequencies obtained from our inferred MaxEnt model f_{ij}^{model} (lower right corner). **B** Associated inferred effective interaction energies ϵ_{ij} (lower right corner, white regions indicate $\epsilon_{ij} \to \infty$) together with a scatter plot of f_{ij}^{expt} vs. f_{ij}^{model} (inset).

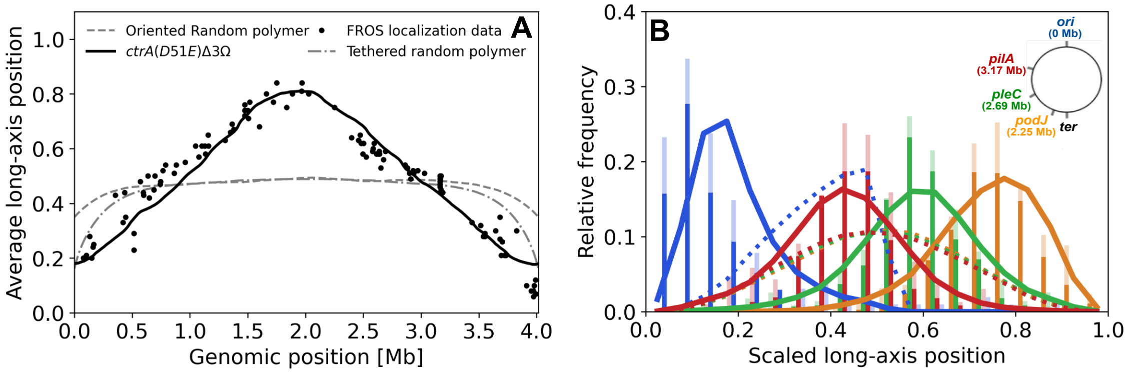

Supplementary Figure 17: Results for Main Text Fig. 2, re-analyzed for the *ctrA(D51E)*$\Delta 3\Omega$ overexpressing cell data set from [15]. **A** Black solid line: average long-axis positions of genomic regions for *ctrA(D51E)*$\Delta 3\Omega$ overexpressing cells predicted by the MaxEnt model. **B** Solid lines: distribution of long-axis positions of chromosomal loci (blue: *ori*, red: *pilA*, green: *pleC*, orange: *podJ*) for *ctrA(D51E)*$\Delta 3\Omega$ overexpressing cells predicted by the MaxEnt model, together with previous experimental data from microscopy experiments (bars, adapted from [3]). To indicate experimental variability, the solid/transparent bars indicate the minimum/maximum measured by two different methods: FROS or FISH.

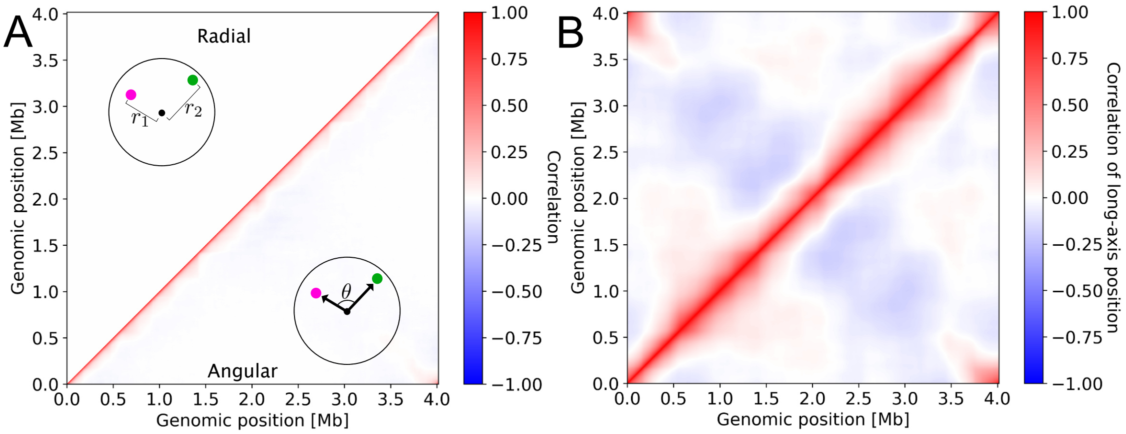

Supplementary Figure 18: Results for Main Text Fig. 3, re-analyzed for the *ctrA(D51E)*$\Delta 3\Omega$ overexpressing cell data set from [15]. **A** Upper left corner: two-point correlations in the radial positions between genomic regions. Lower right corner: two-point correlations in angular orientations around the long axis. **B** Two-point correlations between long-axis positions of genomic regions.

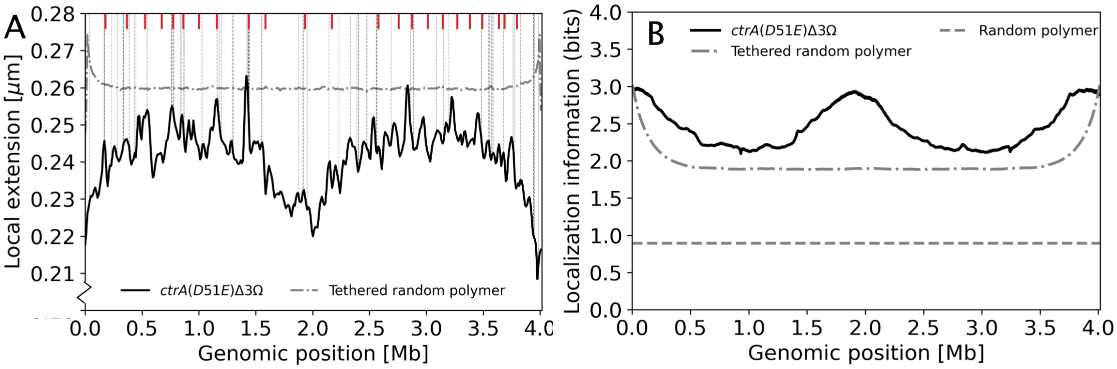

Supplementary Figure 19: Results for Main Text Fig. 5, re-analyzed for the *ctrA(D51E)*Δ*3*Ω overexpressing cell data set from [15]. **A** Black solid line: the local chromosome extension δ_i as a function of genomic position for DnaA-depleted cells as predicted by the MaxEnt model. **B** Black solid line: localization information per genomic region for DnaA-depleted cells as predicted by the MaxEnt model.

9 Analysis of genomic Super Domains

9.1 Super Domain definition and long-axis exclusion analysis

To define genomic Super Domains (SuDs), we first choose a cluster radius r. For each genomic region i, we consider a specific configuration of the chromosome and then calculate the length ℓ of the set of subsequent genomic regions (in both directions along the chromosome) that lie within the radius r from the position of genomic region i (illustrated by the black line in Main Text Fig. 4A). We observe that for each configuration of the chromosome, the genomic regions separate into a small number of domains, indicated by the blue and red areas in Main Text Fig. 4A. We identify a domain with each local maximum in ℓ (indicated by $L1 - L3$ and $R1 - R3$ in Main Text Fig. 4B); the peak location represents the genomic region at the center of a SuD and the peak value indicates the number of genomic regions within the domain.

To determine a natural choice for r, we perform a parameter sweep over r and consider the change in the average value of ℓ with r: $\mathrm{d}\bar{\ell}/\mathrm{d}r$. We find that for the MaxEnt models on wild-type, rifampicin-treated and Δsmc cells, $\mathrm{d}\bar{\ell}/\mathrm{d}r$ initially increases with r, and then becomes approximately constant (Supplementary Fig. 20). For models unconstrained by Hi-C data (the 'random polymer', and the 'tethered random polymer'), such a transition to a plateau regime is not present. We interpret the transition to this plateau regime in the MaxEnt models as the genomic length scale at which the linear organization of the chromosome along the cell length starts dominating local fluctuations of loci(Main Text Fig. 2A&B). We take the crossover point between these two regimes to be $r = 264$ nm, indicated by the grey dashed line in Supplementary Fig. 20.

To quantify the degree of long-axis exclusion between SuDs, the distribution of long-axis positions of the genomic regions contained in each domain is computed (Main Text Fig. 4B). A long-axis position is assigned to a Super Domain based on the highest-occupied long axis coordinate of this cluster. The degree of overlap of long-axis positions is then computed for randomly paired left and right arm configurations and for correctly matched pairs.

9.2 Super Domain properties

To quantify the distribution of SuD sizes and locations, we determined the average number of SuDs on each chromosomal arm, the average SuD size across genomic regions and the distribution of SuD center locations across the genome. The results are shown in Supplementary Figure 21. An illustration of the expected link between SuDs and the inferred anticorrelations between chromosomal arms is shown in Supplementary Figure 22

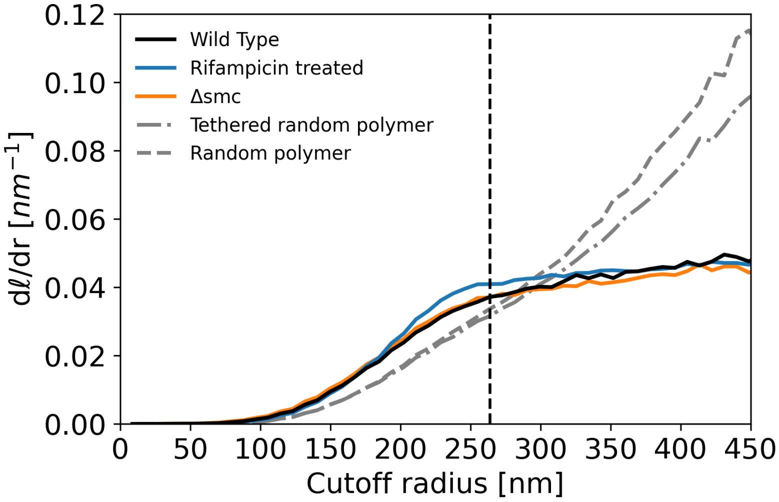

Supplementary Figure 20: Super Domain cluster analysis. Derivative of the average cluster size as a function of the cutoff radius r, for wild-type cells (black), rifampicin-treated cells (blue), a Δsmc mutant (orange), a tethered random polymer (dash-dotted line) and a random polymer (dashed line). The vertical dashed line indicates the chosen cutoff value.

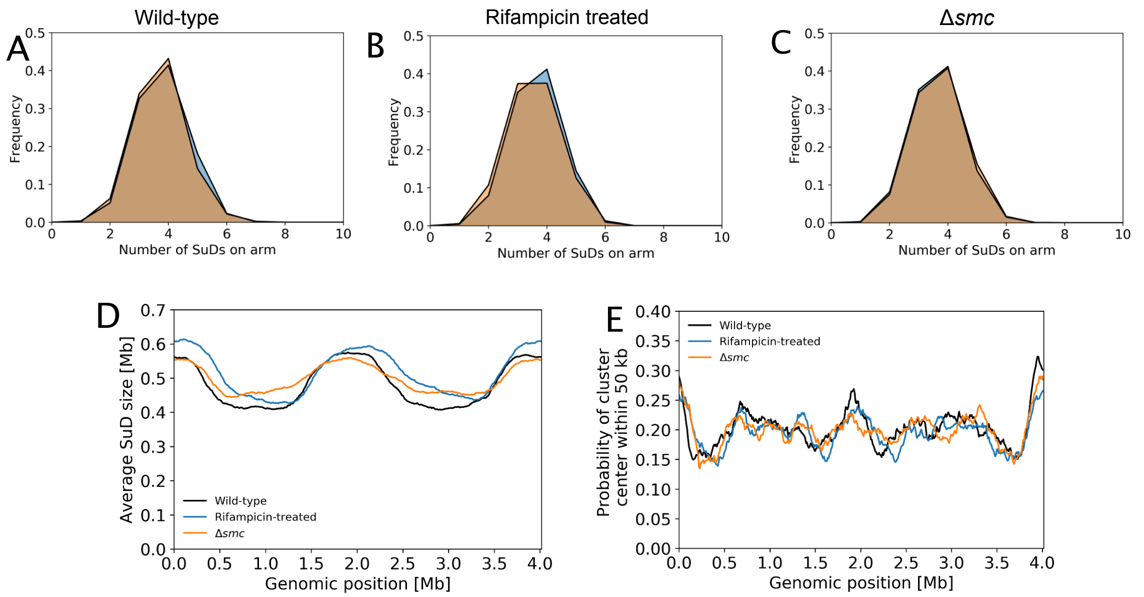

Supplementary Figure 21: Super Domain properties. Distribution of the number of Super Domains across configurations for the left arm (blue) and the right arm (orange) for wild-type cells (**A**), rifampicin-treated cells (**B**) and a Δsmc mutant (**C**). **D** Average size of the SuD a genomic region is part of, given that it is part of a SuD, as a function of genomic position. **E** Probability of a cluster center being within 50 kb of a genomic region, as a function of genomic position.

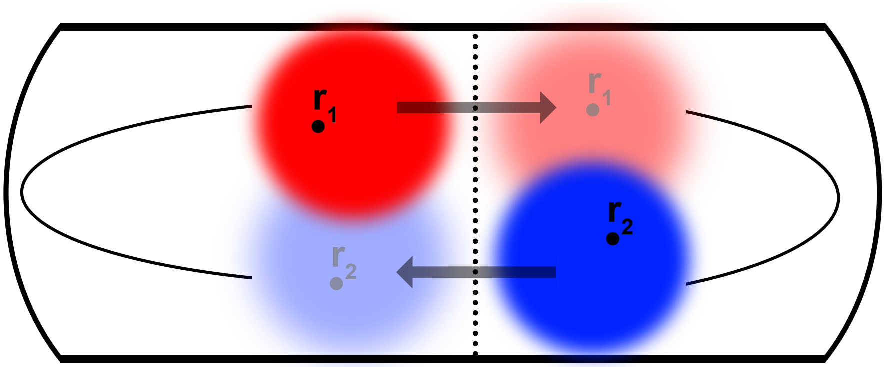

Supplementary Figure 22: Illustration of SuDs inducing anticorrelations between chromosomal arms. In this illustration, genomic regions r_1 and r_2 lie on different chromosomal arms but have the same average long-axis position (dashed line). The SuD that region r_1 is part of, has a tendency to avoid the SuD that region r_2 is part of (given that both regions are part of a SuD). This is expected to induce anticorrelations in the long-axis positions of regions r_1 and r_2.

10 Overlap analysis between local chromosome extension peaks and highly transcribed genes

To investigate the connection between peaks in the local extension profile and the locations of highly transcribed genes, we first construct a (nonlinear) trend line through the chromosome extension profile. This line is constructed by repeatedly applying a Gaussian smoothing filter over the data, incorporating periodic boundary conditions. The Gaussian smoothing is implemented by repeatedly applying a moving average over groups of 3 subsequent genomic regions. We find that 250 repeats to result in a satisfactory balance between smoothing out local peaks and keeping the larger-scale trend (grey line in Supplementary Figure 23A). Next, we select the subset of local extension peaks that lie a factor α above the trend line. We perform a sweep over α and calculate for each choice of α the fraction of incorporated peaks that coincide with the locations of highly transcribed genes. Additionally, for each α we simulate a number of randomly positioned peaks equal to the number of incorporated peaks. From this simulation, we calculate the expected fraction of overlap and the 95% confidence intervals.

We find that the fraction of overlap is significantly higher than expected for randomly positioned local extention peaks, if up to the 9 highest peaks are considered (Supplementary Figure 23B). If more peaks are incorporated, the fraction of overlap gradually decays to the level expected for random positions. Repeating this analysis for the right (0-2 Mb) and left (2-4 Mb) chromosomal arms seperately, we find that the fraction of overlap is only significantly higher than a random guess for the highest peaks of the right arm (Supplementary Figure 23C). For the left arm, by contrast, the fraction of overlap is close to the value expected by random guess for all values of α.

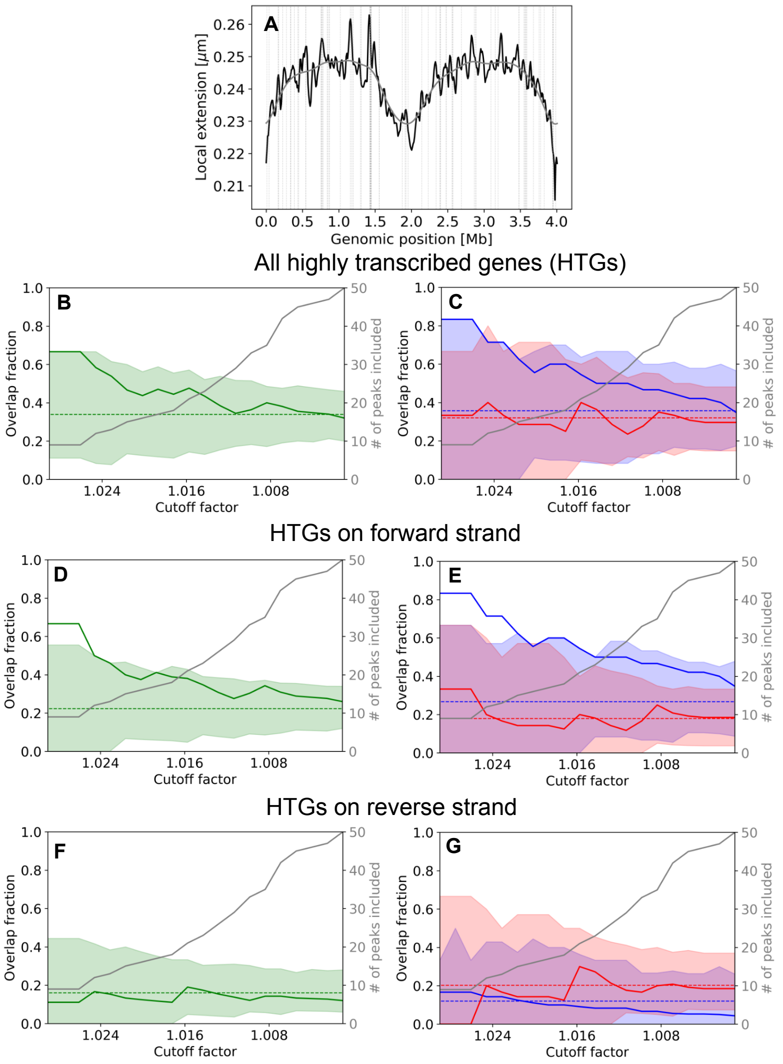

Supplementary Figure 23: Analysis of the degree of overlap between peaks in local chromosome extension and the locations of highly transcribed genes. A Wild-type local chromosome extension profile (black line), together with a trend line obtained from Gaussian smoothing (grey line) and the locations of highly transcribed genes (HTGs) (vertical dashed lines). **B** Green solid line: fraction of local extension peaks that coincide with the location of a highly transcribed gene, as a function of the cutoff factor α. The dashed line indicates the expected fraction of overlap for randomly chosen locations of peaks, the light green area indicates the 95% confidence interval around this expected fraction. The grey line indicates the number of peaks included for a given cutoff factor (indicated on the right axis). **C** The same analysis as in B, performed separately for the right (0-2 Mb, blue) and left (2-4Mb, red) chromosomal arms. **D,E** The same analyses as in B and C, using only the positions of HTGs located on the forward strand of the chromosome. **F,G** The same analyses as in B and C, using only the positions of HTGs located on the reverse strand of the chromosome.

11 Relation between Hi-C scores and average distance and distance correlations

Previous modelling approaches for the *C. crescentus* chromosome used average distance based models to find typical chromosome configurations [13, 14]. In these approaches, an experimentally determined average linear relation between intra-arm genomic distances and average spatial distances was used to derive a functional relation between Hi-C contact scores and average spatial distances. Our MaxEnt model does not require this assumption, instead we can use the model to predict the relation between Hi-C scores and average distances. Interestingly, our MaxEnt model predicts an approximately linear relation between Hi-C scores and average distances, but with significant deviations from this average trend for individual pairs of genomic regions (Supplementary Figure 25A). Moreover, there are substantial deviations from a linear trend for small and large genomic distances. Finally, we also observe significant variations around an average trend for Hi-C scores versus spatial distances (Supplementary Figure 25B).

In addition to these variations in average spatial distances, we also find significant correlations in deviations from these averages for individual configurations throughout the entire chromosome (Supplementary Figure 25). In previously used approaches [13, 14] such correlations could not be taken into account, which could explain the difference in predictions from our MaxEnt model.

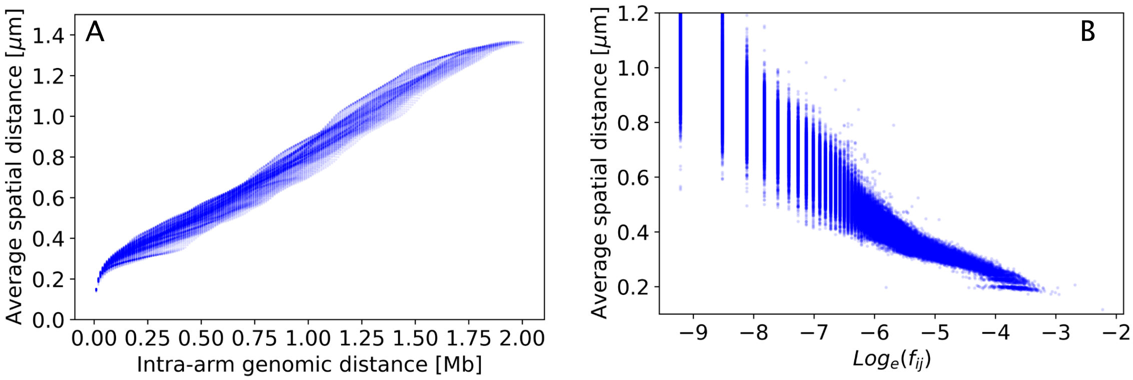

Supplementary Figure 24: Variations of average distance statistics between individual pairs of genomic regions. **A** Average spatial distance versus genomic distance predicted by the MaxEnt model. **B** Average spatial distance versus the logarithm of the Hi-C score predicted by the MaxEnt model.

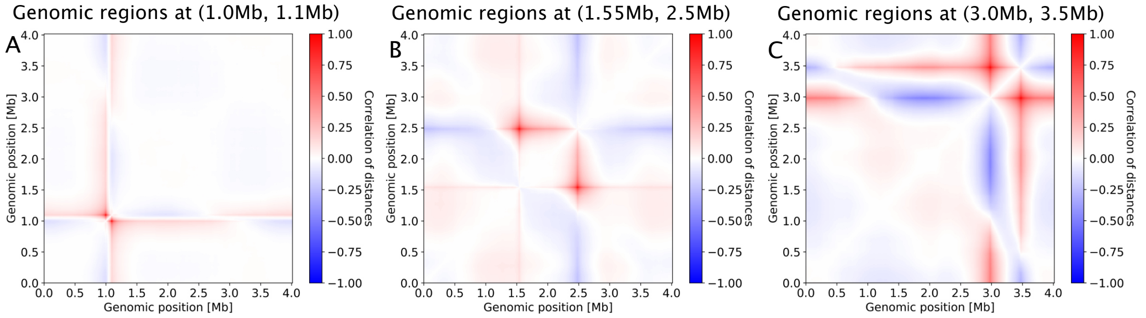

Supplementary Figure 25: Correlations between distances of all pairs of genomic regions, and the distance between three sample pairs. The chosen sample pairs are: **A** genomic regions at (1.0Mb, 1.1Mb), **B** genomic regions at (1.55Mb, 2.5Mb), **C** genomic regions at (3.0Mb, 3.5Mb).

12 A global rotation does not produce the observed long-axis correlation pattern

To illustrate the features of a long-axis correlation map that would be induced by a global rotation, we simulated the effects of such rotational fluctuations. Specifically, we took a set of configurations from our model, and generated an ensemble of new configurations by performing a rotational fluctuation with a random magnitude of all genomic regions along the polymers axial coordinate within each configuration. The magnitude of this rotation was drawn from a zero-average normal distribution, with the standard deviation σ treated as a free parameter. For this new ensemble of configurations, including global rotation fluctuations, the long-axis correlations were calculated between all genomic regions. The resulting long-axis correlation maps for this rotational model for four choices of the standard deviation are shown in Supplementary Figure 26.

We see that for $\sigma = 0.2Mb$, the magnitude of correlations in the rotation model (Supplementary Figure 26A, upper left) is comparable to those observed in the original MaxEnt model (Main Text Fig. 3B, upper left). Importantly however, the anticorrelations in the rotation model are present between all genomic regions on opposite stretches of the chromosome. Thus, in this case, we see anti-correlation both between opposing genomic regions on the left and right chromosome arm and between opposing genomic regions near *ori* and *ter*. This is in contrast to the pattern observed in the original MaxEnt model, where the anticorrelations are only present between juxtaposed genomic regions lying on opposite sides of the left and right chromosome arms and opposing genomic regions near *ori* and *ter* exhibit positive correlations (Main Text Fig. 3B, upper left). For larger values of σ, the anticorrelation pattern in the rotation model initially remains qualitatively the same as for low σ, but the magnitude of correlations increases (Supplementary Figure 26A, lower right). For even larger values of σ, the long-axis correlation pattern starts to qualitatively change: the region of anticorrelation between *ori* and *ter* becomes larger (Supplementary Figure 26B). Furthermore, the magnitude of anticorrelations is much higher for these values of σ than observed in the original MaxEnt model.

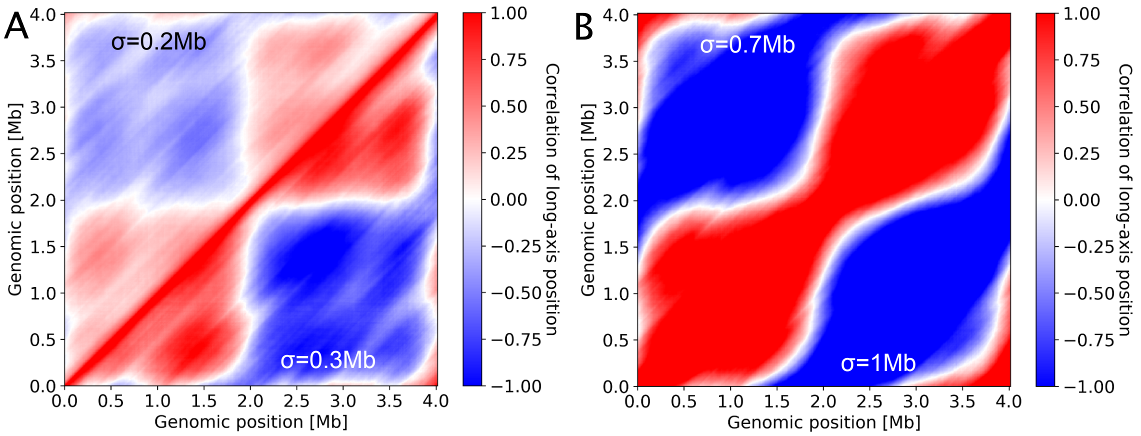

Supplementary Figure 26: Long-axis correlations for chromosome configurations with global rotational fluctuations. A) Upper left: long-axis correlations for model configurations with global rotational fluctuations along the polymer axis, drawn from a normal distribution with σ=0.2Mb. Lower right: the same for σ=0.3Mb. B) Upper left: same for σ=0.7Mb, lower right: same for σ=1Mb.

13 MaxEnt models for Δ*smc* cells and rifampicin-treated cells

We apply the same approach to perform a Hi-C data analysis and MaxEnt model inference for rifampicin-treated cells and Δ*smc* cells. The prepossessing of Hi-C data is shown in Figs. 27 and 28, and the corresponding MaxEnt models are shown in Figs. 29 and 30. We show the results for the long-axis localization in Supplementary Figure 31 together with previously published experimental data, and various correlation functions are depicted in Supplementary Figure 32.

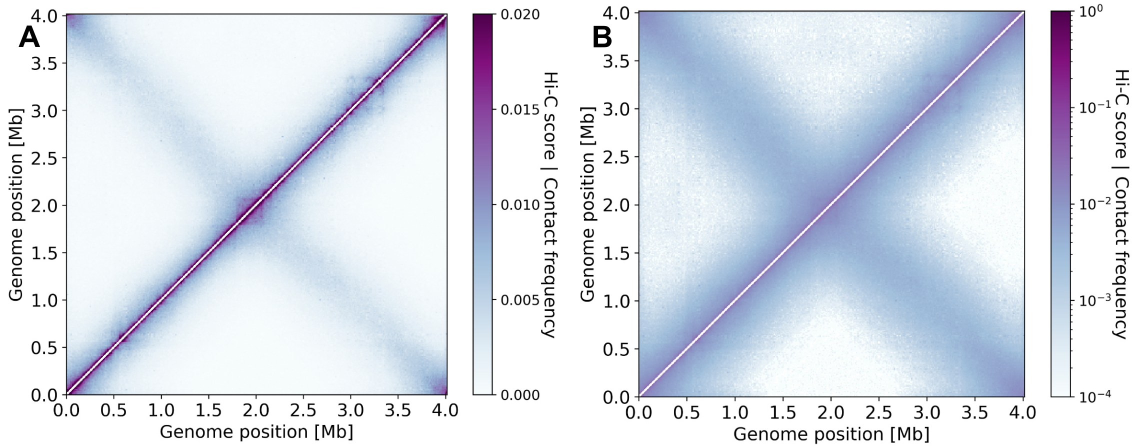

Supplementary Figure 27: Hi-C scores for rifampicin-treated cells before and after correction. Hi-C scores of rifampicin-treated cells before correction (upper left triangle), and after correction (lower right triangle) on a linear scale (A) and a logarithmic scale (B).

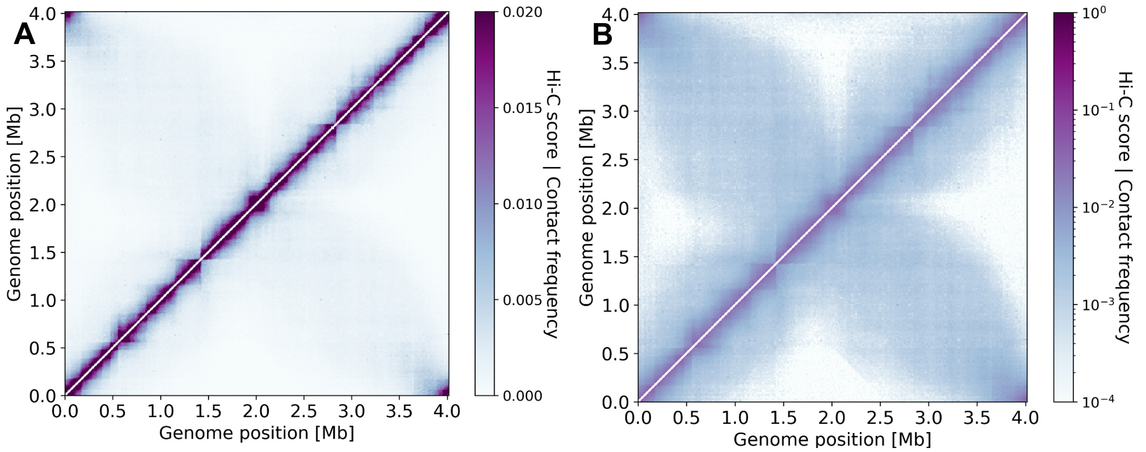

Supplementary Figure 28: Hi-C scores for Δsmc cells before and after correction. Hi-C scores of Δsmc cells before correction (upper left triangle), and after correction (lower right triangle) on a linear scale (A) and a logarithmic scale (B).

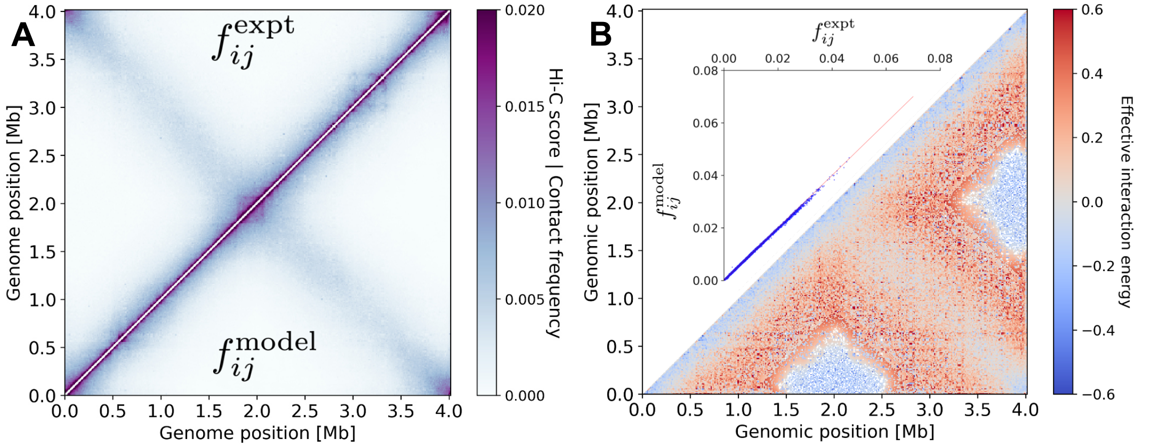

Supplementary Figure 29: Maximum entropy model inferred for rifampicin-treated cells. **A** Comparison between experimental contact frequencies f_{ij}^{expt} (upper left corner, adapted from Ref. [12]) and contact frequencies obtained from our inferred MaxEnt model f_{ij}^{model} (lower right corner). **B** Inferred effective interaction energies ϵ_{ij} (lower right corner) together with scatterplot of f_{ij}^{expt} vs. f_{ij}^{model} (inset).

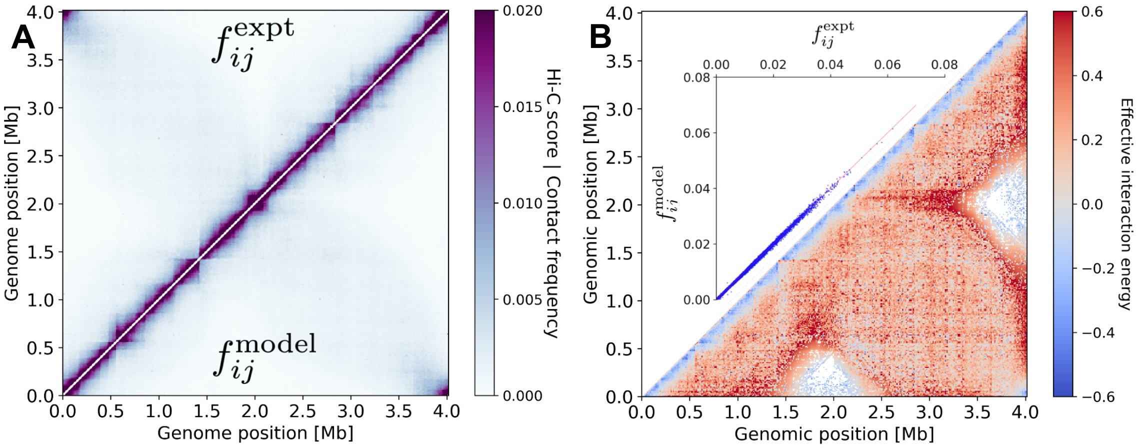

Supplementary Figure 30: Maximum entropy model inferred for Δ*smc* cells. A comparison between experimental contact frequencies $f_{ij}^{\rm expt}$ (upper left corner, adapted from Ref. [12]) and contact frequencies obtained from our inferred MaxEnt model $f_{ij}^{\rm model}$ (lower right corner). **B** Inferred effective interaction energies ϵ_{ij} (lower right corner) together with scatterplot of $f_{ij}^{\rm expt}$ vs. $f_{ij}^{\rm model}$ (inset).

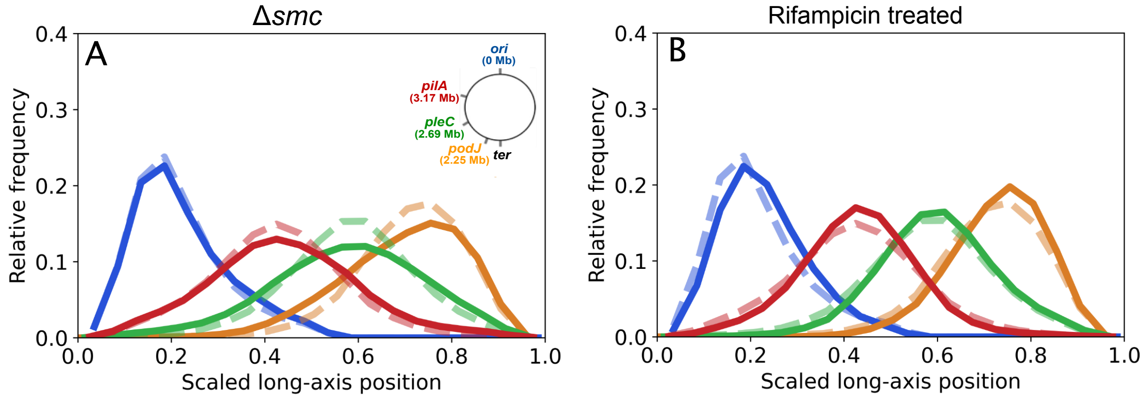

Supplementary Figure 31: Distribution of long-axis positions for Δ*smc* and rifampicin-treated cells. Comparison between inferred long-axes localization distributions for wild-type cells (dashed lines) and Δ*smc* mutants (**A**, solid lines) and rifampicin-treated cells (**B**, solid lines).

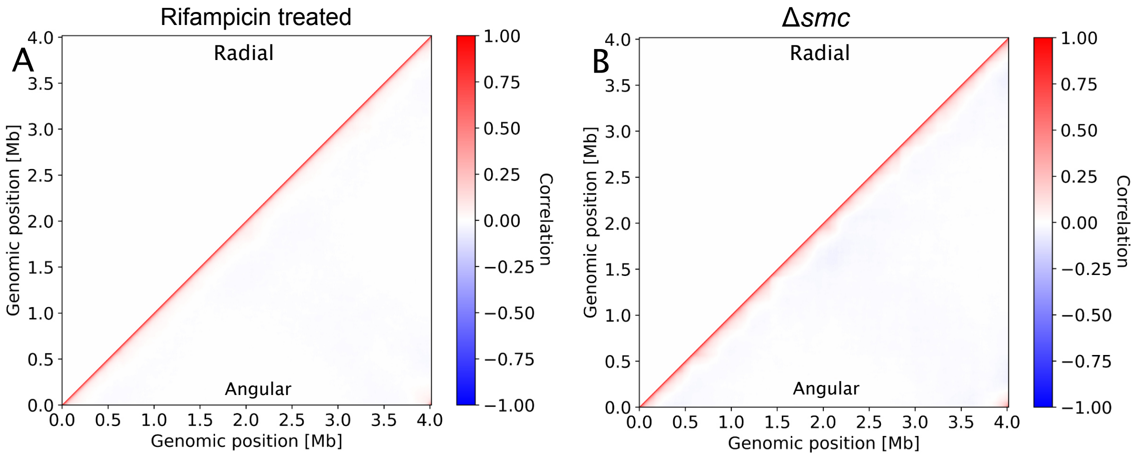

Supplementary Figure 32: Radial and angular correlations for Δ*smc* and rifampicin-treated cells. Correlations in the radial positions (upper left corner) and orientations around the long axis (lower right corner) between all pairs of genomic regions, for rifampicin-treated cells (**A**) and Δ*smc* mutants (**B**).

14 Estimates of localization information

To compute the localization information for a genomic region, we first calculate the average occupation $P^{s,i}$ of each unit cell s for each genomic region i during a forward simulation. The localization entropy S^i_{loc} in bits of site i is then calculated by [16]

$$S^i_{\text{loc}} = -\sum_s P^{s,i} \log_2 P^{s,i}. \tag{S8}$$

The positional information is calculated by subtracting S^i_{loc} from the localization entropy of a flat distribution.

A possible issue with calculating positional information within a coarse-grained model, is that the obtained value is an underestimate. This is the case if the localization is confined to a region approximately the size of a unit cell. Since we find the localizations of genomic regions to be significantly larger than this (Main Text Fig. 2B), we do not expect our estimate to be sensitive to the course graining scale.

15 Local extension interval and origin of *ori* and *ter* extensions

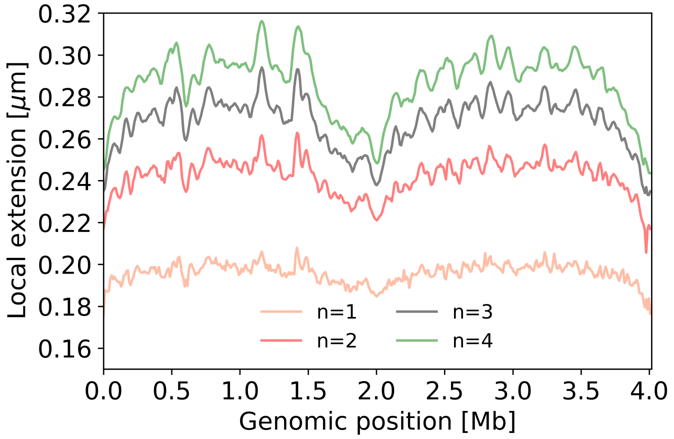

Supplementary Figure 33: Change of local extension with genomic distance Local extensions, defined as the average distance between the nth nearest neighbours of a genomic region, shown for $n = 1$ up to $n = 4$. The value of $n = 2$ is shown in the main text as its features are more prominent than those for $n = 1$, but less smoothened out than for higher values of n. The locations of the peaks are largely identical between these different choices for n.

A possible explanation for the low local extension of the *ori* and *ter* regions, would be the turning around of the average long-axis positions at these regions. As the local extension of a region is calculated as the average geometric distance between its nth neighbours, such an effect could cause the observed low local extension. To test if this is the case, we make use of the presence

of variations in the positions of the *ori* and *ter*; for a subset of states, these will not be the furthest regions along the long axis. If the inferred low local extension is indeed due to a 'turning around' of the chromosome at the *ori* and *ter*, the local extension would be expected to be higher for this subset of states.

Taking a conditional average of the local extension of the *ori* over states where the previous 5 or subsequent 5 genomic regions all have an equal or lower long-axis position than the *ori* region, we find an increase of only 2% compared to an average over all states. For the *ter* region, we find the same statistics (2% increase if either set of 5 neighboring regions has a higher or equal long-axis position than the *ter*). Thus, the inferred local density of the *ori* and *ter* regions reflect the intrinsic extensions of these regions, rather than artefacts due to a turning around of the average long axis positions at these sites.

16 Linear spatial organization of a polymer with juxtaposed chromosomal arms

To investigate organizational features of a polymer with juxtaposed arms, but no additional structure, we derive a MaxEnt model taking average long-axis positions as the only constraints. This model we term the linearly organized polymer model. To enable a direct comparison to MaxEnt models learned from Hi-C data, we take the average long-axis positions predicted from these models and use these as constraints for the linearly organized polymer. This allows us to investigate to what extent features of the MaxEnt model based on Hi-C data are due to its linear organization throughout the cell. For the linearly organized polymer model, the entropy functional takes the following form:

$$\tilde{S} = -\sum_{\{\mathbf{r}\}} P(\{\mathbf{r}\}) \ln P(\{\mathbf{r}\}) - \sum_i \lambda_i \left(\sum_{\{\mathbf{r}\}} P(\{\mathbf{r}\}) z_i(\mathbf{r}) \quad - \langle z_i \rangle^{\text{cons}} \right) - \lambda_0 \left(\sum_{\{\mathbf{r}\}} P(\{\mathbf{r}\}) - 1 \right). \tag{S9}$$

Here, $z_i(\mathbf{r})$ denotes the long-axis position of region i in configuration $\mathbf{r}$, and $\langle z_i \rangle^{\text{cons}}$ denotes the imposed average long-axis position of region i. Extremizing this entropy functional and solving for $P(\{\mathbf{r}\})$ yields

$$P(\{\mathbf{r}\}) = \frac{1}{Z} \exp \left[-\sum_i \lambda_i z_i(\mathbf{r}) \right], \tag{S10}$$

with $Z = \exp[1 + \lambda_0]$ as in the main text. The solutions for λ_i were found with an iterative Monte Carlo algorithm similar to the one presented in 3, where the update of λ_i at each iteration of the inverse algorithm is now proportional to $\langle z_i \rangle^{\text{cons}} - \langle z_i \rangle^{\text{model}}$. The resulting organizational properties of the linearly organized polymer model are presented in Supplementary Figure 34.

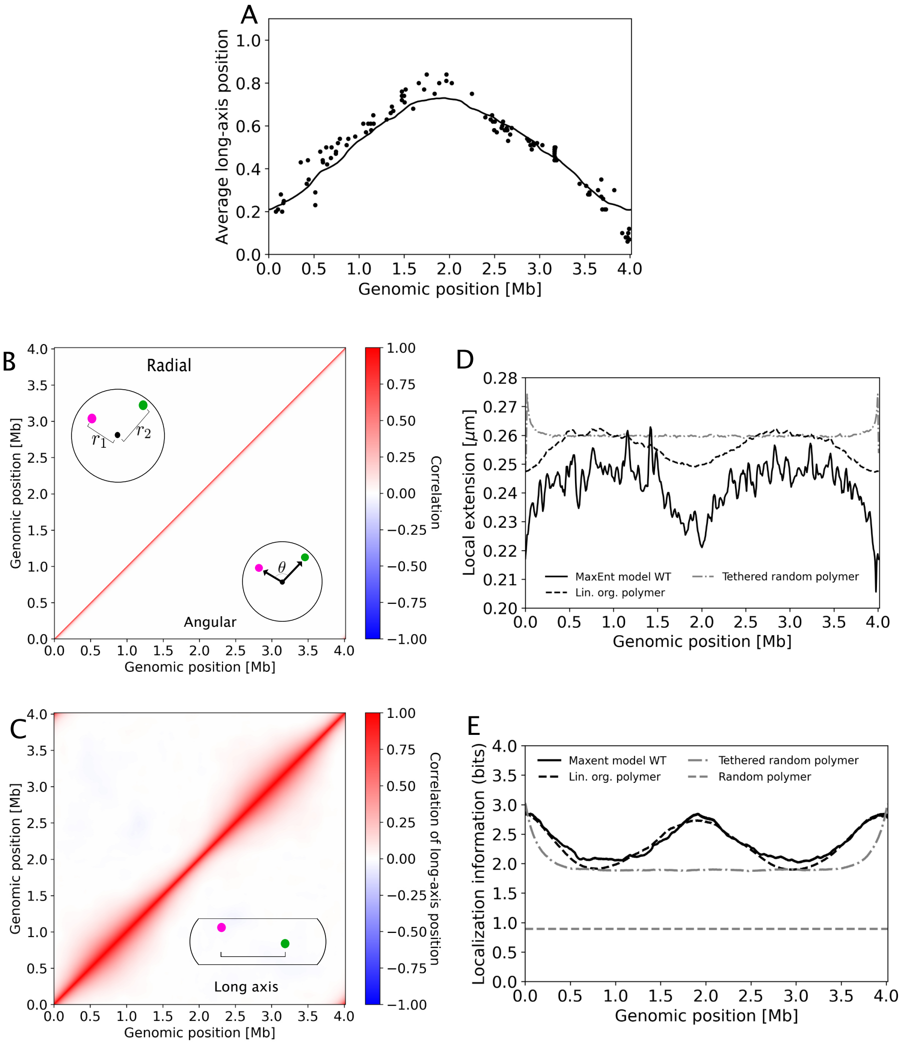

Supplementary Figure 34: Model results for the linearly organized polymer model. A) Average localization profile used as a constraint of the linearly organized polymer (line) together with the experimental FISH [3] data shown in Main Text Fig. 2A. (dots). **B**)) Radial correlations (upper left triangle) and angular correlations (lower right triangle) for the linearly organized polymer. **C**)) Long-axis correlations for the linearly organized polymer. **D**)) Results for the local extension as in Main Text Fig. 5A, together with those for the linearly organized polymer. **E**)) Results for the localization information as in Main Text Fig. 5B, together with those for the linearly organized polymer.

17 Independence of results for modified MaxEnt models

To test if our results are robust under minor model modifications, we inferred two alternative MaxEnt models: one with a slightly curved confinement, and one with a tethered *ori*. The former incorporates the typically observed *C. crescentus* cell shape, the latter enforces the experimentally measured long-axis distribution of the position of the *ori* locus. The inferred models are shown in Figs. 36 and 37.

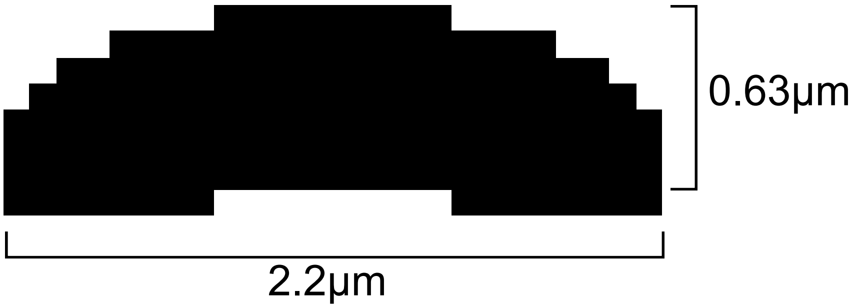

Supplementary Figure 35: Top view of the curved cell shape used for analyses presented in this Note. A lattice spacing corresponds to 88nm, as in the Main Text model.

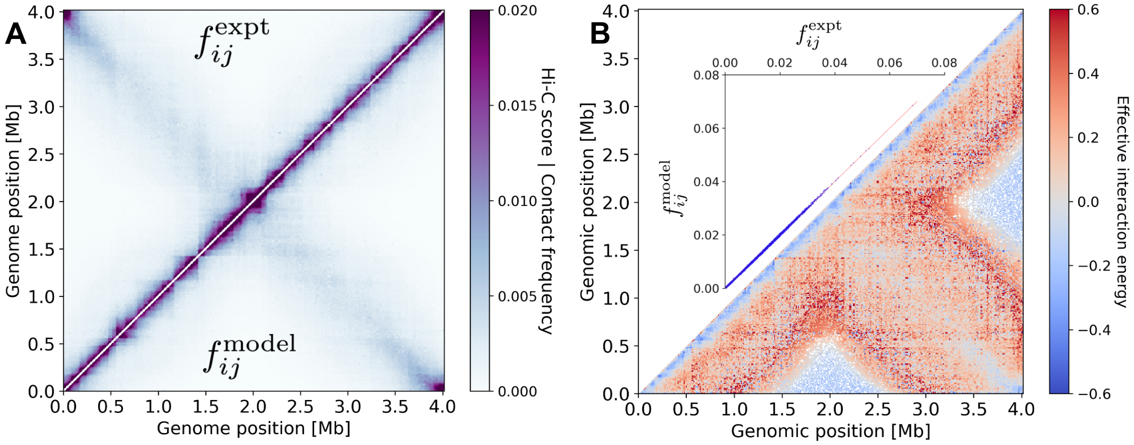

Supplementary Figure 36: Results for Main Text Fig. 1, re-analyzed for a model with tethered *ori*. **A** Comparison between experimental contact frequencies f_{ij}^{expt} (upper left corner, adapted from Ref. [12] and contact frequencies obtained from our inferred MaxEnt model f_{ij}^{model} (lower right corner). **B** Associated inferred effective interaction energies ϵ_{ij} (lower right corner, white regions indicate $\epsilon_{ij} \to \infty$) together with a scatter plot of f_{ij}^{expt} vs. f_{ij}^{model} (inset).

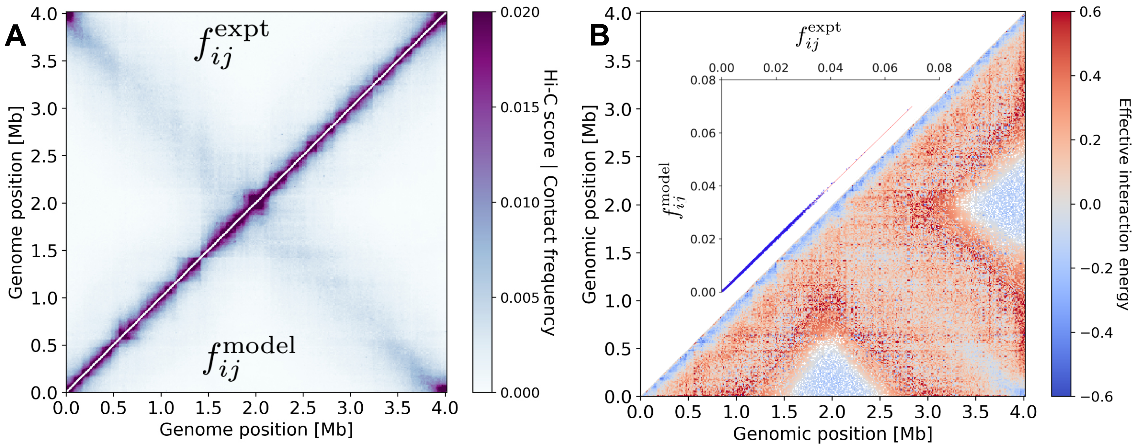

Supplementary Figure 37: Results for Main Text Fig.1, re-analyzed for a model with a curved cell A Comparison between experimental contact frequencies f_{ij}^{expt} (upper left corner, adapted from Ref. [12] and contact frequencies obtained from our inferred MaxEnt model f_{ij}^{model} (lower right corner). **B** Associated inferred effective interaction energies ϵ_{ij} (lower right corner, white regions indicate $\epsilon_{ij} \to \infty$) together with a scatter plot of f_{ij}^{expt} vs. f_{ij}^{model} (inset).

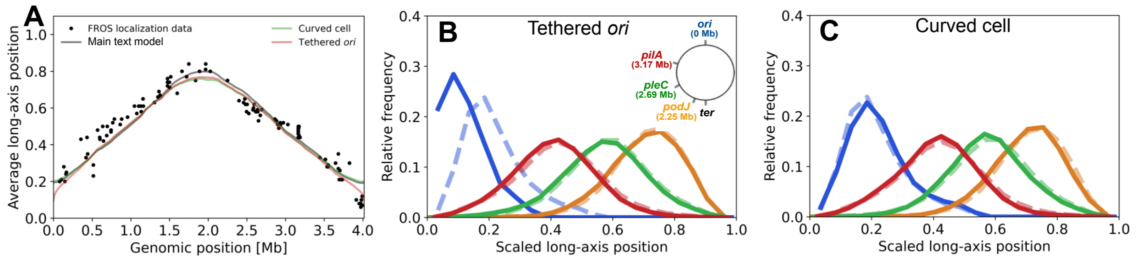

Supplementary Figure 38: Results for Main Text Fig. 2, re-analyzed for a model with a tethered *ori* and a curved cell. A Average scaled long-axis position predicted from MaxEnt models (solid lines) inferred for various MaxEnt models, including the model described in the main text (black), a model for a curved cell (green), and a model with a tethered *ori* (red), together with results from microscopy experiments (adapted from [3]). **B** Solid lines: localizations for a MaxEnt model with a tethered *ori*. Dashed lines: Maxent model results as presented in Main Text Fig. 2. **C** Solid lines: localizations for a MaxEnt model with a curved cell. Dashed lines: Maxent model results as presented in Main Text Fig. 2.

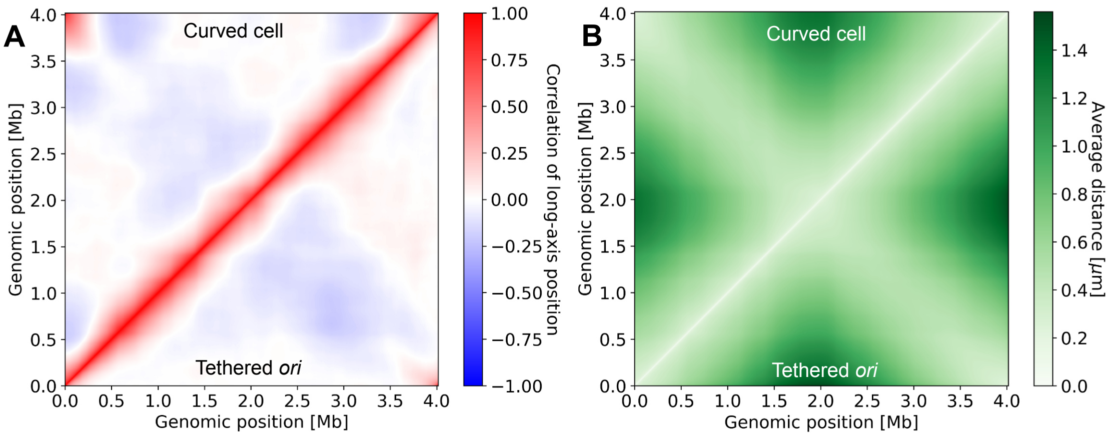

Supplementary Figure 39: Long-axis correlations and average distances for Maxent models with a curved cell and a tethered *ori*. **A** Long-axis correlations for a Maxent model with a curved cell (top left) and a tethered *ori* (bottom right). **B** Average distances for a Maxent model with a curved cell (top left) and a tethered *ori* (bottom right).

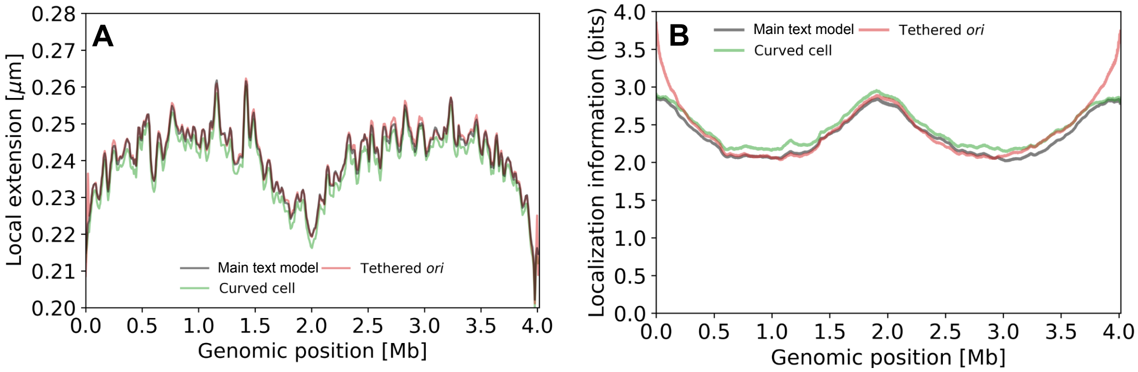

Supplementary Figure 40: Results for Main Text Fig. 5, re-analyzed for a model with a tethered *ori* and a curved cell. **A** The local chromosome extension δ_i as a function of genomic position. Model prediction are shown for the model described in the main text (black), a model for a curved cell (green), and a model with a tethered *ori* (red). **B** Localization information per genomic region in bits for the model described in the main text (black), a model for a curved cell (green), and a model with a tethered *ori* (red).

References

[1] Lau, I. F. *et al.* Spatial and temporal organization of replicating *Escherichia coli* chromosomes. *Mol. Microbiol.* **49**, 731–743 (2004).

[2] Ely, B. Genetics of *Caulobacter crescentus*. *Methods in Enzymology* **204**, 372–384 (1991).

[3] Viollier, P. H. *et al.* Rapid and sequential movement of individual chromosomal loci to specific subcellular locations during bacterial DNA replication. *Proc. Natl. Acad. Sci. USA* **101**, 9257–9262 (2004).

[4] Tsai, J. W. & Alley, M. R. Proteolysis of the *Caulobacter* McpA chemoreceptor is cell cycle regulated by a ClpX-dependent pathway. *J. Bacteriol.* **183**, 5001–5007 (2001).

[5] University of Sussex. Image J Analysis: Single-molecule plugins (17.01.2020).

[6] Rueden, C. T. *et al.* ImageJ2: ImageJ for the next generation of scientific image data. *BMC Bioinformatics* **18**, 529 (2017). arXiv:1701.05940.

[7] Evinger, M. & Agabian, N. Envelope associated nucleoid from *Caulobacter crescentus* stalked and swarmer cells. *J. Bacteriol.* **132**, 294–301 (1977).

[8] Thanbichler, M., Iniesta, A. A. & Shapiro, L. A comprehensive set of plasmids for vanillate and xylose-inducible gene expression in *Caulobacter crescentus*. *Nucleic Acids Res.* **35**, e137–e137 (2007).

[9] De Martino, D., MC Andersson, A., Bergmiller, T., Guet, C. C. & Tkačik, G. Statistical mechanics for metabolic networks during steady state growth. *Nature Communications* **9**, 2988 (2018).

[10] Hartmann, R., Van Teeseling, M. C., Thanbichler, M. & Drescher, K. Bacstalk: a comprehensive and interactive image analysis software tool for bacterial cell biology. *Molecular Microbiology* (2020).

[11] Gan, L., Chen, S. & Jensen, G. J. Molecular organization of Gram-negative peptidoglycan. *Proc. Natl. Acad. Sci. USA* **105**, 18953–18957 (2008).

[12] Le, T. B. K., Imakaev, M. V., Mirny, L. A. & Laub, M. T. High-resolution mapping of the spatial organization of a bacterial chromosome. *Science* **342**, 731–734 (2013).

[13] Umbarger, M. A. *et al.* The three-dimensional architecture of a bacterial genome and its alteration by genetic perturbation. *Mol. Cell* **44**, 252–264 (2011).

[14] Yildirim, A. & Feig, M. High-resolution 3D models of *Caulobacter crescentus* chromosome reveal genome structural variability and organization. *Nucleic Acids Res.* **46**, 3937–3952 (2018).

[15] Le, T. B. & Laub, M. T. Transcription rate and transcript length drive formation of chromosomal interaction domain boundaries. *EMBO J.* **35**, 1582–1595 (2016).

[16] Dubuis, J. O., Tkacik, G., Wieschaus, E. F., Gregor, T. & Bialek, W. Positional information, in bits. *Proc. Natl. Acad. Sci. USA* **110**, 16301–16308 (2013).
