# Supplementary material for: Learning the distribution of single-cell chromosome conformations in bacteria reveals emergent order across genomic scales
Source: Nat Commun. 2021 Mar 30;12:1963. doi: 10.1038/s41467-021-22189-x (PMC8010069; doi:10.1038/s41467-021-22189-x)
Supplement: Supplementary file 3 — Reporting Summary [file 41467_2021_22189_MOESM3_ESM.pdf]

nature research

Corresponding author(s): Chase P. Broedersz

Last updated by author(s): Jan 17, 2021

Reporting Summary

Nature Research wishes to improve the reproducibility of the work that we publish. This form provides structure for consistency and transparency in reporting. For further information on Nature Research policies, see our Editorial Policies and the Editorial Policy Checklist.

Statistics

For all statistical analyses, confirm that the following items are present in the figure legend, table legend, main text, or Methods section.

n/a	Confirmed	
☐	☒	The exact sample size (*n*) for each experimental group/condition, given as a discrete number and unit of measurement
☐	☒	A statement on whether measurements were taken from distinct samples or whether the same sample was measured repeatedly
☐	☒	The statistical test(s) used AND whether they are one- or two-sided<br>*Only common tests should be described solely by name; describe more complex techniques in the Methods section.*
☐	☒	A description of all covariates tested
☐	☒	A description of any assumptions or corrections, such as tests of normality and adjustment for multiple comparisons
☐	☒	A full description of the statistical parameters including central tendency (e.g. means) or other basic estimates (e.g. regression coefficient) AND variation (e.g. standard deviation) or associated estimates of uncertainty (e.g. confidence intervals)
☒	☐	For null hypothesis testing, the test statistic (e.g. *F*, *t*, *r*) with confidence intervals, effect sizes, degrees of freedom and *P* value noted<br>*Give P values as exact values whenever suitable.*
☒	☐	For Bayesian analysis, information on the choice of priors and Markov chain Monte Carlo settings
☐	☒	For hierarchical and complex designs, identification of the appropriate level for tests and full reporting of outcomes
☐	☒	Estimates of effect sizes (e.g. Cohen's *d*, Pearson's *r*), indicating how they were calculated

Our web collection on statistics for biologists contains articles on many of the points above.

Software and code

Policy information about availability of computer code

Data collection	For microscopy: VisiView 2.1.4 (Visitron), For super-resolution microscopy: ZEN 3.0 SR Black Edition(Zeiss)
Data analysis	For microscopy: to measure cell lengths: BacStalk version 1.7 stable, to measure localization of fluorescently labeled loci (at subpixel accuracy): GDSC SMLM plugin for ImageJ2. For the rest: custom algorithms.

For manuscripts utilizing custom algorithms or software that are central to the research but not yet described in published literature, software must be made available to editors and reviewers. We strongly encourage code deposition in a community repository (e.g. GitHub). See the Nature Research guidelines for submitting code & software for further information.

Data

Policy information about availability of data

All manuscripts must include a data availability statement. This statement should provide the following information, where applicable:

- Accession codes, unique identifiers, or web links for publicly available datasets
- A list of figures that have associated raw data
- A description of any restrictions on data availability

The datasets generated during and/or analysed during the current study are available from the corresponding authors on reasonable request. We provide a sample of chromosome configurations generated by our Maximum Entropy model on GitHub.

Field-specific reporting

Please select the one below that is the best fit for your research. If you are not sure, read the appropriate sections before making your selection.

☒ Life sciences ☐ Behavioural & social sciences ☐ Ecological, evolutionary & environmental sciences

For a reference copy of the document with all sections, see nature.com/documents/nr-reporting-summary-flat.pdf

Life sciences study design

All studies must disclose on these points even when the disclosure is negative.

Sample size	For distances between loci: 6 different strains (corresponding to 5 independent pairs of loci) were analyzed, and for each strain 100 cells were analyzed. The error margins for each individual strain, as well as for the average over all strains, are detailed in supplementary table S5. The sample sizes were chosen to ensure sufficient accuracy in the determination of the average distance between loci. The final error margin of 7nm (SEM) on the average distance between the loci is indeed small enough for the purposes of our model.
Data exclusions	Cells that were clearly larger than a new-born swarmer cell (length 2.3 +- 0.2 um) were not taken into account during measurement of the distances between loci. All measurements were used for the analysis. For SuD visualization (Fig. 4D), displayed cells were selected on the same length criterion.
Replication	For measurements concerning distances between loci: the replication consisted of creating two strains, MvT171 and MvT172, for the same loci pairs, but with the markers interchanged (SI Section S1). The deviation of inferred average distances for the two strains are within error margin (SI Table S5).<br>For super-resolution microscopy the experiment was replicated and the findings could be reproduced.<br>No further replication experiments were performed, thus all replication attempts were successful.
Randomization	Not applicable for our study. For SIM microscopy: Only one condition was studied, therefore no randomization of samples was possible. For microscopy measurements of distance between loci: all 6 strains (each with two loci fluorescently tagged) were generated from the same parental strain. As each strain was used as a specific group, no further randomization was possible.
Blinding	Not applicable for our study. For SIM microscopy: Only one condition was studied, therefore no blinding was possible. For analysis of microscopy images to determine the distance between loci: a strong protocol for inclusion of cells in the analysis was made before analysis was started (see paragraph data exclusion). This protocol was strictly adhered to, leaving no room for bias introduced by the researcher.

Reporting for specific materials, systems and methods

We require information from authors about some types of materials, experimental systems and methods used in many studies. Here, indicate whether each material, system or method listed is relevant to your study. If you are not sure if a list item applies to your research, read the appropriate section before selecting a response.

Materials & experimental systems

n/a	Involved in the study	
☒	☐	Antibodies
☒	☐	Eukaryotic cell lines
☒	☐	Palaeontology and archaeology
☐	☒	Animals and other organisms
☒	☐	Human research participants
☒	☐	Clinical data
☒	☐	Dual use research of concern

Methods

n/a	Involved in the study	
☒	☐	ChIP-seq
☒	☐	Flow cytometry
☒	☐	MRI-based neuroimaging

Animals and other organisms

Policy information about studies involving animals; ARRIVE guidelines recommended for reporting animal research

Laboratory animals	No animals, only the bacterial species Caulobacter crescentus was used.
Wild animals	NA
Field-collected samples	NA
Ethics oversight	NA

Note that full information on the approval of the study protocol must also be provided in the manuscript.
